# Decanoic acid extends lifespan and modulates metabolism in models of *PLA2G6*-associated neurodegeneration

**DOI:** 10.1242/dmm.052184

**Published:** 2025-12-04

**Authors:** Biqin Zhang, Ella Dunn, Robin S. B. Williams, Stuart Snowden, Hrvoje Augustin

**Affiliations:** Centre for Biomedical Sciences, Department of Biological Sciences, Royal Holloway University of London, Egham, Surrey TW20 0EX, UK

**Keywords:** Neurodegenerative disease, Decanoic acid, Medium-chain fatty acid, PLAN, *Drosophila*

## Abstract

*PLA2G6*-associated neurodegeneration (PLAN) is a group of rare genetic disorders characterised by progressive neurodegeneration resulting from mutations in the *PLA2G6* gene, encoding a calcium-independent phospholipase enzyme. Here, we have explored the effects of decanoic acid (DA), a medium-chain fatty acid, in the fruit fly *Drosophila melanogaster* models of PLAN and show that DA treatment significantly extends the lifespan, reduces bang sensitivity and improves resistance to heat shock stress. Transcriptional analysis showed that DA affects genes in key signalling pathways, including Insulin/Insulin-like Growth Factor, mTOR, heat shock response, Sirtuin, autophagy and mitochondrial function. Additionally, DA treatment alters the metabolite profiles in PLAN model flies, with the most pronounced changes observed in gut tissue. Pathway analysis of these metabolomic shifts highlights potential therapeutic effects of DA in several pathways, including ATP-binding cassette (ABC) transporters, purine metabolism, cAMP signalling and neuroactive ligand-receptor interactions. These findings suggest that DA may be a promising therapeutic agent for PLAN, offering insights into the mechanisms of the disease and paving the way for future research on medium-chain fatty acids as potential treatments for neurodegenerative diseases.

## Teaser

**Summary:** Decanoic acid, a medium-chain fatty acid, extends lifespan and improves the response to some forms of stress in *Drosophila* models of a rare neurodegenerative disease.

## INTRODUCTION

*PLA2G6*-associated neurodegeneration encompasses a group of rare, recessively inherited disorders caused by loss-of-function mutations in the *PLA2G6* gene with an estimated prevalence of 1:1,000,000 (Gregory et al., 1993; Morgan et al., 2006; Guo et al., 2018; Deng et al., 2023; Hanna Al-Shaikh et al., 2022; Hinarejos et al., 2020). The *PLA2G6* gene encodes a group VI calcium-independent phospholipase A2 enzyme (iPLA2β) found at the plasma membrane, Golgi apparatus, nuclear envelope, mitochondria and the endoplasmic reticulum (Seleznev et al., 2006; Deng et al., 2023; Fais et al., 2021). The iPLA2β catalyses the cleavage of sn-2 acyl-ester bonds in glycerophospholipids, producing free fatty acids and lysophospholipids (Ma and Turk, 2001; Wolf and Gross, 1996). This enzyme plays a vital role in phospholipid membrane remodelling, and these products serve as precursors for lipid mediators involved in cellular signalling (Sumi-Akamaru et al., 2015; Lubrano et al., 2023; Kano et al., 2022).

Mitochondrial dysfunction has been implicated in both human PLAN patients and animal PLAN models, including the fruit-fly *Drosophila melanogaster* and mice (Beck et al., 2011; Kinghorn et al., 2015; Lin et al., 2018). Mutations in *PLA2G6* lead to the accumulation of abnormal phospholipids, disrupting mitochondrial structure and function. This results in impaired mitochondrial dynamics, defective oxidative phosphorylation, increased reactive oxygen species (ROS) production, reduced ATP levels and activation of apoptosis (Beck et al., 2011; Paradies et al., 2019; Ke et al., 2020). Mouse models with nonfunctional iPLA2β exhibit reduced mitochondrial function in the brain (Beck et al., 2011) and impaired autophagy (Zhou et al., 2016). Notably, pharmacological enhancement of lysosomal and mitochondrial function reduces neurodegeneration in both PLAN model flies and patient-derived cells (Iliadi et al., 2018). PLAN models in *Drosophila* were created by introducing loss-of-function mutations in the *iPLA2-VIA* gene, which is the fly homolog of *PLA2G6* (Kinghorn et al., 2015; Lin et al., 2018; Banerjee et al., 2021; Mori et al., 2019). These flies exhibit widespread neuronal degeneration throughout the brain, loss of dopaminergic neurons, mitochondrial damage, motor dysfunction, reduced lifespan, decreased female fertility, synaptic transmission defects at the neuromuscular junction and photoreceptor impairment (Kinghorn et al., 2015; Lin et al., 2018; Banerjee et al., 2021; Mori et al., 2019).

Decanoic acid, also known as capric acid, is a medium-chain fatty acid (MCFA) that has been explored for its potential therapeutic effects in various neurological conditions (Chang et al., 2016; Włodarek, 2019). DA and other MCFAs have been studied in the context of the ketogenic diet (Chang et al., 2013, 2015), which has proven effective as an alternative treatment for drug-resistant epilepsy (Li et al., 2024). Owing to their chemical properties, including solubility, lipophilicity and affinity for carrier-mediated transporters, MCFAs, unlike long-chain fatty acids, can readily cross the blood–brain barrier (Ebert et al., 2003; Johnson et al., 1990; Spector, 1988). DA has also been associated with reduced inflammation, which is a key contributor to neurodegeneration in many diseases (Huang et al., 2014a). Chronic brain inflammation can exacerbate neuronal damage (Glass et al., 2010; Frank-Cannon et al., 2009), so the anti-inflammatory properties of DA could offer significant therapeutic benefits. Additionally, DA treatment has been shown to enhance mitochondrial complex I activity and increase mitochondrial numbers, supporting neuronal health (Hughes et al., 2014; Pain et al., 2024). DA has also been suggested to stimulate brain-derived neurotrophic factor (BDNF) production (Sharma et al., 2023), which promotes neuron growth and survival, playing a crucial role in maintaining neuronal health and potentially slowing the progression of neurodegenerative diseases like Alzheimer's disease (AD) (Miranda et al., 2019; Gao et al., 2022).

Despite these promising mechanisms, more research is needed to fully understand the effects of DA on neurodegeneration in the context of PLAN. This study aims to evaluate the impact of DA in two *Drosophila* models of PLAN. Our findings provide evidence that DA improves several PLAN-related phenotypes, including reduced lifespan, impaired heat stress resistance, neuronal dysfunction and metabolic imbalances.

## RESULTS

### DA treatment extends the lifespan in *Drosophila* PLAN models

**Fig. 1.**
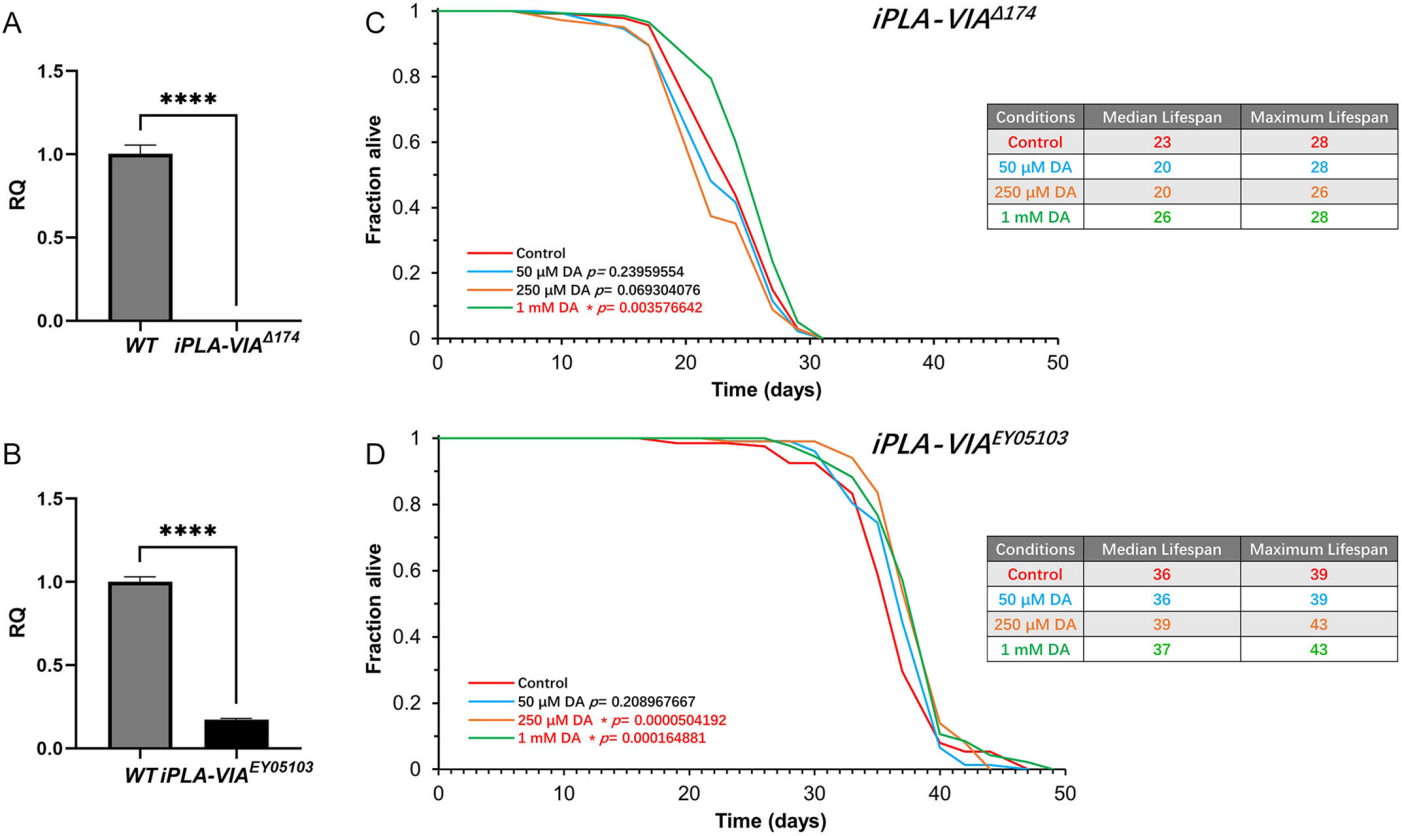
**DA extends the lifespan in both *Drosophila* PLAN models.** (A) qPCR analysis of *iPLA-VIA* expression levels in wild-type (WT) and *iPLA-VIA^Δ174^* flies (*n*=3). (B) Validation of *iPLA-VIA* expression levels in WT and *iPLA-VIA^EY05103^* flies (*n*=3). (C) Lifespan of *iPLA-VIA^Δ174^* flies is significantly longer when the flies are fed food containing 1 mM DA (*n*=150). (D) The lifespan of *iPLA-VIA^EY05103^* flies is significantly extended when they are fed 250 μM and 1 mM DA (*n*=150). Statistical significance in A and B was determined using an unpaired, two-tailed *t*-test (****P<0.0001). RQ represent relative expression level. Data in C and D were analysed using the log-rank test, with *P*-values comparing the given DA concentration to control. Lifespan is presented as survival curves, showing the percentage of surviving flies over time. Each experiment was repeated at least twice with similar results (Fig. S2).

We first examined the effect of DA on the lifespan of two mutant lines lacking *iPLA2-VIA* expression: *iPLA-VIA^Δ174^* and *iPLA2-VIA^EY05103^* (Kinghorn et al., 2015; Lin et al., 2018). To validate the PLAN models flies, we performed qPCR analysis, which revealed no detectable *iPLA-VIA* expression in *iPLA-VIA^Δ174^* flies (Fig. 1A) and significantly reduced *iPLA-VIA* expression in *iPLA2-VIA^EY05103^* flies (Fig. 1B). Consistent with previous studies (Kinghorn et al., 2015; Lin et al., 2018; Iliadi et al., 2018; Banerjee et al., 2021), lifespan analysis demonstrated that *iPLA-VIA^Δ174^* flies had a shorter lifespan than *iPLA2-VIA^EY05103^* flies (Fig. 1C,D). Lifespan assays were then conducted on flies fed with 50 μM, 250 μM or 1 mM DA. The *iPLA-VIA^Δ174^* flies showed a significant increase in median lifespan at the highest DA concentration (1 mM) (Fig. 1C). While treatment with 50 μM and 250 μM DA resulted in a slight reduction in the median lifespan of *iPLA-VIA^Δ174^*, this reduction was not statistically significant (Fig. 1C). In contrast, *iPLA2-VIA^EY05103^* flies showed significant lifespan extension at both 250 μM and 1 mM DA (Fig. 1D). Together, these findings highlight the longevity-promoting effects of DA in both *Drosophila* PLAN models.

### No effect of DA on locomotion or climbing ability

We then aimed to explore the impact of DA on healthspan to determine whether the observed lifespan extension is accompanied by improvements in overall health and functional capacity. To evaluate healthspan, we performed climbing and locomotion assays on DA-treated PLAN flies, assessing motor function and physical activity levels as indicators of health. The results showed no significant differences in either motor performance or physical activity between the DA-treated flies and their controls (Fig. S3A-D). These findings suggest that, while DA extends lifespan, it does not seem to enhance key healthspan markers in the context of PLAN.

### DA reduces bang sensitivity in *iPLA-VIA^Δ174^* flies

Some *Drosophila* mutants experience stress-sensitive seizures and paralysis when subjected to mechanical stimulation, a phenomenon referred to as bang-sensitivity (Ganetzky and Wu, 1982; Pavlidis and Tanouye, 1995; Kuebler and Tanouye, 2000). This condition is typically observed in flies with compromised mitochondrial function and is frequently linked to lifespan and brain degeneration (Sen and Cox, 2017; Celotto et al., 2006; Burman et al., 2014). The ‘bang test’ in *Drosophila* serves as a model for investigating the genetic and molecular mechanisms of epilepsy, which can be a symptom of adult-onset dystonia-Parkinsonism, a subtype of PLAN (Guo et al., 2018). The *iPLA-VIA^Δ174^* mutants have been documented to exhibit an age-dependent increase in recovery time from mechanical stimuli, along with abnormal mitochondrial morphology (Lin et al., 2018).

**Fig. 2.**
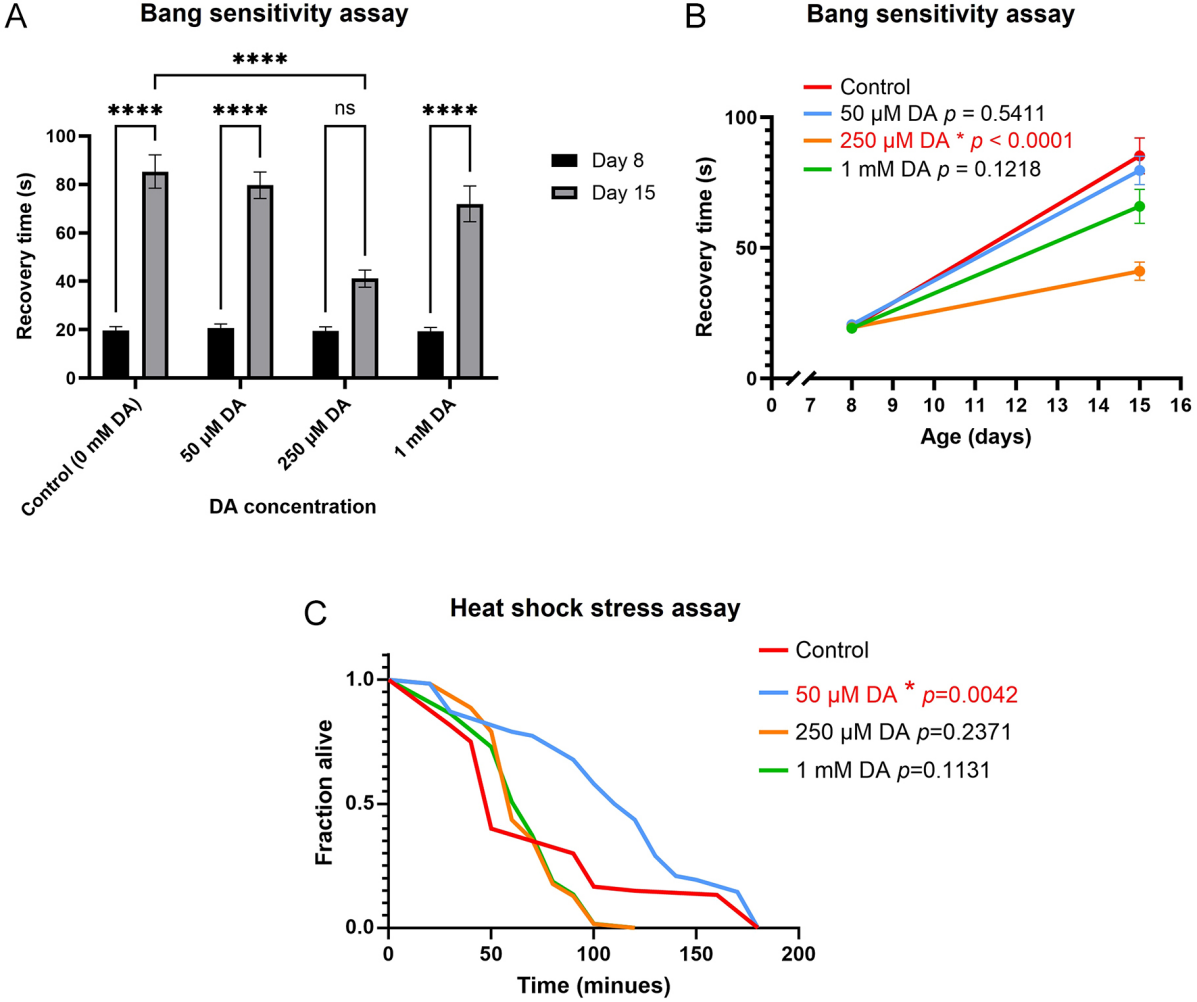
**DA reduces bang sensitivity and improves heat-shock stress resistance of *iPLA-VIA^Δ174^* flies.** (A) Bang-sensitivity assay in *iPLA-VIA^Δ174^* flies at days 8 and 15 post-eclosion, fed with 50 μM, 250 μM and 1 mM DA. Flies treated with DMSO (control), 50 μM and 1 mM DA showed a significant increase in recovery time from mechanical stress-induced paralysis between day 8 and day 15 (*P<*0.0001), whereas flies fed 250 μM DA did not show a significant change (*P*=0.0609) (*n*=50-75). (B) Treatment with 250 μM DA significantly slowed the rate of increase (slope) of recovery time in *iPLA-VIA^Δ174^* flies compared to control flies (*P<*0.0001) (*n*=50-75). (C) Heat-shock stress resistance assay in young (3-day-old) *iPLA-VIA^Δ174^* flies fed DA at different concentrations (50 μM, 250 μM and 1 mM). Flies fed 50 μM DA (blue line) exhibited significantly improved heat-shock stress resistance compared to the controls (red line) (*n*=150). In A, statistical analysis was performed using two-way ANOVA with Tukey's multiple comparisons test in GraphPad Prism. In B, a simple linear regression analysis was used in GraphPad Prism. In C, data were analysed using the log-rank test. Differences were considered significant at *P*<0.05. Error bars represent s.e.m. ****P<0.0001; n.s., not significant. The bang-sensitivity assay was repeated at least twice with similar results.

To explore this further, a bang-sensitivity test was conducted on both young (day 8) and old (day 15) *iPLA-VIA^Δ174^* flies fed varying concentrations of DA (50 μM, 250 μM, and 1 mM). The recovery time was significantly longer in day 15 flies treated with DMSO, 50 μM and 1 mM DA compared to day 8 flies (Fig. 2A), indicating an age-related increase in bang sensitivity, consistent with previous studies (Lin et al., 2018). Interestingly, the age-dependent increase in recovery time was significantly reduced in older flies treated with 250 μM DA (Fig. 2A), resulting in a slower rate of increase in bang sensitivity between days 8 and 15 (Fig. 2B). These results highlight the potential of DA as a therapeutic agent for combating age-related neuronal dysfunction and suggest it may help mitigate the effects of mitochondrial impairment in this model.

### DA improves heat-shock stress resistance

In model organisms, increased stress resistance is often linked to extended lifespans (Soo et al., 2023a,b; Bhat et al., 2024). Additionally, reduced resistance to oxidative and starvation stress has been observed in *Drosophila* PLAN models (Lin et al., 2018; Iliadi et al., 2018). Although heat-shock resistance has not been previously studied in *Drosophila* PLAN models, decreased resistance to thermal stress has been reported in other fly models of neurodegenerative diseases, such as AD (Tsakiri et al., 2021). To investigate the effects of DA further, we assessed stress resistance in DA-treated flies. After 3 days of treatment with different DA concentrations, the flies underwent a heat-shock stress assay. A lower dose of DA (50 μM) significantly improved heat shock resistance in young *iPLA-VIA^Δ174^* flies (Fig. 2C), suggesting an acute effect of the compound. However, DA did not enhance heat resistance in older flies (data not shown), likely due to a decline in their stress-response mechanisms that prevented a protective effect. Additionally, DA did not affect resistance to starvation or oxidative stress (Fig. S3E,F), nor did it influence the activity of superoxide dismutase (SOD) or catalase, key antioxidant enzymes involved in ageing and longevity (Fig. S3G,H). These findings suggest that DA specifically and selectively improves heat-shock resistance in young PLAN flies, and that its lifespan-extending effect is independent of enzymatic antioxidant defences. Given the difference in the concentrations of DA that extend lifespan and protect against heat shock (50 µM and 1 mM, respectively), our data imply that the compound likely acts through distinct mechanisms or pathways at different concentrations, with low doses optimising acute stress resistance and higher doses promoting long-term effects such as lifespan extension.

### Expression profiling of genes in *iPLA-VIA^Δ174^* flies treated with DA

The *iPLA-VIA^Δ174^* flies exhibited a more severe phenotype, with a greater reduction in lifespan compared to *iPLA2-VIA^EY05103^* flies (Kinghorn et al., 2015; Lin et al., 2018). 1 mM DA treatment had a stronger effect on *iPLA-VIA^Δ174^* flies, extending lifespan by 13%, compared to ∼8% in *iPLA-VIA^EY05103^* flies (Fig. 1B,D). To further explore the impact o DA on ageing in the PLAN model, we examined the expression of genes involved in key pathways and processes related to lifespan regulation and stress response in *iPLA-VIA^Δ174^* flies. These included anabolic pathways such as Insulin/Insulin-like Growth Factor (IGF) signalling (IIS) and Target of Rapamycin (TOR), as well as the sirtuin pathway, autophagy and mitochondrial function.

**Fig. 3.**
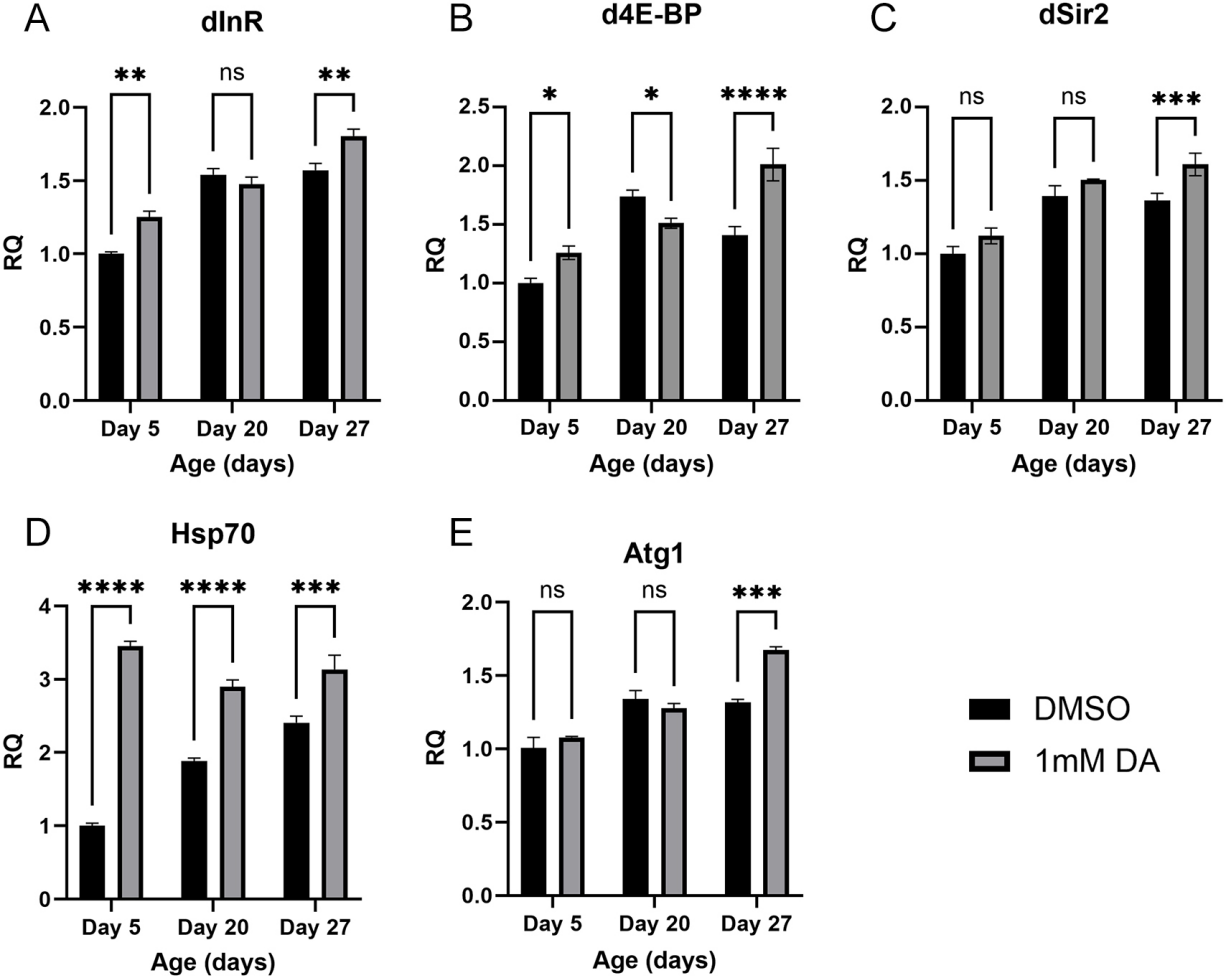
**DA modulates the expression of key genes involved in insulin signalling, sirtuin regulation, heat-shock response and autophagy initiation.** (A-E) In *iPLA-VIA^Δ174^* flies fed 1 mM DA or control food (DMSO): (A) *InR* expression is increased at days 5 and 27 compared to age-matched controls (treatment/age interaction: *P*=0.0035); (B) *4E-BP* expression is elevated at days 5 and 27 but reduced at day 20 (****P<0.0001); (C) *Drosophila Sirt1* (dSir2) expression is increased at day 27, although the treatment/age interaction is not significant (*P*=0.1069); (D) *Hsp70* expression is elevated across all time points (****P<0.0001); (E) *Atg1* expression is increased at day 27 (*P*=0.0009). Statistical analyses of all RQ values were performed using two-way ANOVA with Tukey's multiple comparisons test in GraphPad Prism. Error bars represent s.e.m. Significance is indicated as *P<0.0332, **P<0.0021, ***P<0.0002, ****P<0.0001; n.s., not significant (*n*=3).

The IIS and TOR pathways are central regulators of growth, metabolism and lifespan (Giannakou and Partridge, 2007; Partridge et al., 2011). In the IIS pathway, *Drosophila Insulin receptor* (*InR*) acts as an upstream regulator (Grönke et al., 2010), while the transcription factor FOXO serves as its downstream effector (Hwangbo et al., 2004). The *Drosophila* 4E-binding protein (4E-BP) is a downstream target of the TOR pathway (Carvalho et al., 2017), and overexpression of either *foxo* or *4E-BP* (*Thor*) in *Drosophila* tissues significantly extends lifespan (Demontis and Perrimon, 2010; Giannakou et al., 2004). DA treatment resulted in an age-related increase in *InR* and *4E-BP* expression (Fig. 3A,B), without a corresponding change in *foxo* expression (Fig. S4A).

The *Drosophila Silent information regulator 2* (*Sir2*; officially known as *Sirt1*) plays a crucial role in the sirtuin pathway (Frankel et al., 2011), which regulates stress resistance, energy metabolism and lifespan (Frankel et al., 2011). Loss of *Sirt1* results in a shortened lifespan (Aström et al., 2003), while its overexpression promotes longevity (Frankel et al., 2011). DA treatment increased *Sirt1* expression on day 27 (Fig. 3C), suggesting that its lifespan-extending effects may involve age-associated activation of the sirtuin pathway. Heat-shock protein 70 (Hsp70) is a molecular chaperone that supports protein homeostasis and enhances stress resilience (Evans et al., 2010; Feder and Hofmann, 1999); its upregulation has been linked to increased stress resistance and extended lifespan (Zhao et al., 2005). DA treatment significantly elevated *Hsp70* expression at all ages (Fig. 3D), suggesting improved protein homeostasis and a potential mechanism through which the compound exerts its effects.

Autophagy, a process that degrades and recycles cellular components (Mizushima and Komatsu, 2011), plays a crucial role in neurodegenerative diseases (Aman et al., 2021; Giorgi et al., 2021) and is associated with increased longevity and oxidative resistance when upregulated (Simonsen et al., 2008; Nakamura and Yoshimori, 2018; Gelino and Hansen, 2012; Madeo et al., 2015). We examined the expression of genes involved in different stages of autophagy, including pre-autophagy, initiation, autophagosome formation, elongation and closure, autophagosome-lysosome fusion, and lysosomal enzyme activity. We found that only *Atg1* showed increased expression after DA treatment, suggesting that DA influences the initiation but not the overall rate of autophagy (Fig. 3E, Fig. S5A).

Atg8a is essential for autophagosome formation, and its loss disrupts autophagy, leading to developmental defects, muscle impairment and neurodegeneration (Mizushima, 2020; Xu et al., 2024). Ref2, the *Drosophila* homolog of p62/SQSTM1, accumulates in protein aggregates when autophagy is impaired and is linked to longevity and healthspan extension (Nezis et al., 2008; Aparicio et al., 2019). In *Drosophila*, Atg8a and Ref2 levels are commonly used as markers of autophagy activity (Nagy et al., 2015). However, western blot analysis of these key autophagy-related proteins showed no effect of DA treatment (Fig. S5B-E). Since mitochondrial dysfunction is a prominent feature of *Drosophila* PLAN models (Beck et al., 2011; Kinghorn et al., 2015), we also examined the expression of genes related to mitochondrial function, dynamics and biogenesis. DA treatment had no significant effect on the expression of these genes (Fig. S4B), suggesting a limited impact on mitochondrial function. In summary, our gene expression analysis suggests that DA modulates the IIS/TOR pathway, enhances the sirtuin pathway and heat shock response, and promotes autophagy initiation without affecting its overall rate.

### DA alters metabolite profiles in *Drosophila* PLAN models

To explore the underlying mechanism of the effects of DA on lifespan in PLAN model flies and to assess DA-induced metabolic alterations, we conducted metabolomic analyses of three key tissues - the brain, gut and muscle – as well as whole flies. A panel of 42 identified metabolites from 23 distinct metabolic pathways was measured and analysed in both PLAN models (*iPLA-VIA^EY05103^* and *iPLA-VIA^Δ174^*) treated with 250 µM and 1 mM DA, respectively.

**Fig. 4.**
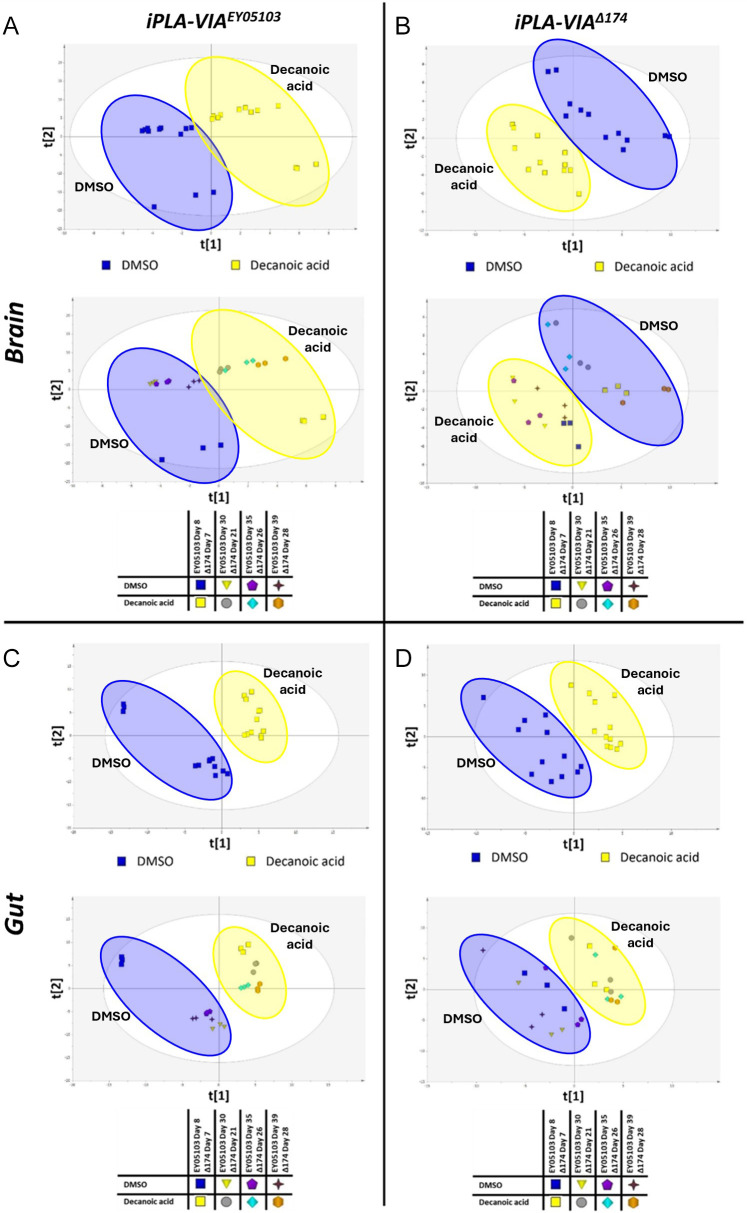
**PLS-DA scores plots showing the effect of ageing and DA treatment of metabolite profile in brain and gut.** Ellipses identify respective treatment groups, with blue indicating DMSO-treated and yellow indicating DA-treated samples. (A) PLS-DA score plots coloured by treatment and age in the brain of *iPLA-VIA^EY05103^* flies (R^2^X=0.627 R^2^Y=0.809 Q^2^=0.505 CV-ANOVA=6.42×10^−7^). (B) PLS-DA score plots coloured by treatment and age in the brain of *iPLA-VIA^Δ174^* flies (R^2^X=0.543 R^2^Y=0.517 Q^2^=0.752 CV-ANOVA=5.43×10^−5^). (C) PLS-DA score plots coloured by treatment and age in the gut of *iPLA-VIA^EY05103^* flies (R^2^X=0.553 R^2^Y=0.919 Q^2^=0.882 CV-ANOVA=6.61×10^−8^). (D) PLS-DA scores plots coloured by treatment and age in the gut of *iPLA-VIA^Δ174^* flies (R^2^X=0.274 R^2^Y=0.862 Q^2^=0.787 CV-ANOVA=0.0001) (*n*=3).

Initial multivariate analysis using PLS-DA revealed that DA treatment had a significant impact on metabolism across all tissues in both *iPLA-VIA^EY05103^* and *iPLA-VIA^Δ174^* flies, with the most pronounced effects observed in the brain and gut (Fig. 4). This analysis also indicated that ageing strongly influenced metabolic function in *iPLA-VIA^EY05103^* flies (Fig. 4A,C), whereas in *iPLA-VIA^Δ174^* flies, the metabolic profile remained relatively stable throughout the lifespan.

**Fig. 5.**
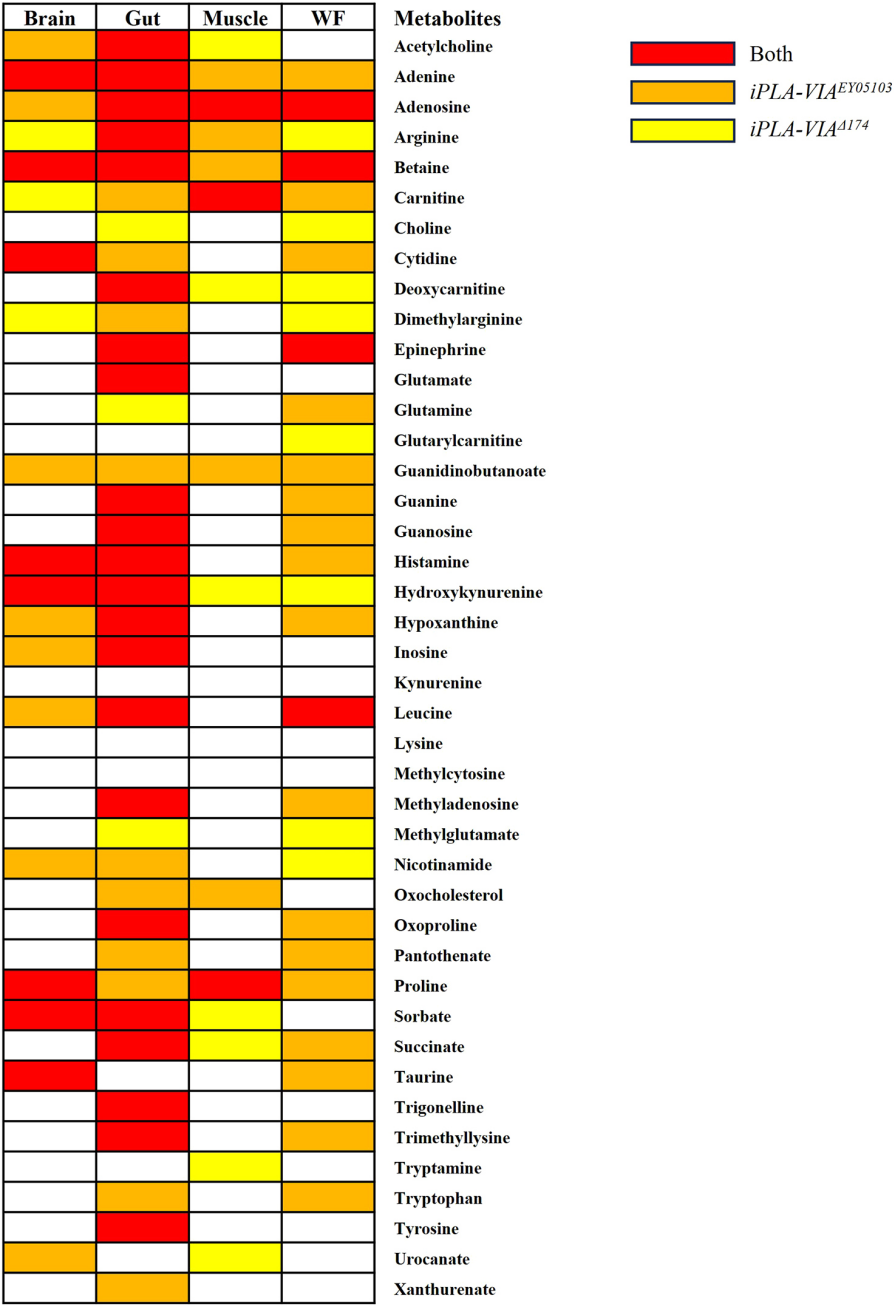
**Heat map of metabolites altered by DA in *Drosophila* PLAN models.** The heat map provides an overview of changes in the relative abundance of individual metabolites detected across various tissues, including the brain, gut, muscle and whole flies, from two *Drosophila* PLAN models treated with either DMSO (control) or 250 μM DA. Metabolites are indicated based on their pattern of alteration: those highlighted in red represent metabolites that showed a significant (*P<*0.05) interaction between DA treatment and age across both PLAN models; those in orange indicate metabolites affected exclusively in the *iPLA-VIA^EY05103^*; and those in yellow indicate metabolites affected exclusively in the *iPLA-VIA^Δ174^*. The results showed a pronounced effect of DA in the gut tissue, where the highest number of shared metabolic alterations across both PLAN models was observed. Statistical analysis was conducted using a two-way ANOVA with Tukey's multiple comparisons tests in GraphPad Prism for each metabolite in each tissue sample. The treatment/age interaction is highly significant (*P<*0.0001) (*n*=3).

Metabolites with a significant interaction between treatment and age in both PLAN models were highlighted in red, while those specifically affected in *iPLA-VIA^EY05103^* or *iPLA-VIA^Δ174^* flies are marked in orange and yellow, respectively (Fig. 5). The levels of certain metabolites fluctuated across different time points, with the figure highlighting those significantly altered by DA, regardless of whether their levels increased or decreased. Notably, the gut exhibited the highest number of DA-modulated metabolites shared between both PLAN models, suggesting a conserved metabolic response to DA in this tissue.

### Metabolic pathway analysis reveals key targets of DA in *Drosophila* PLAN models

Across all four sample types – brain, gut, muscle and whole flies – and both studied genotypes, 218 structurally distinct metabolites were robustly measured, annotated and passed quality control screening. Initially, we looked to determine if there was a significant overlap in the metabolic signature associated with the interaction between DA and ageing observed in *iPLA-VIA^EY05103^* and *iPLA-VIA^Δ174^*. A total of 8, 22, 3 and 4 overlapping metabolites were found in the brain, gut, muscle and whole-fly samples, respectively. To determine whether these overlaps were due to chance or reflected underlying biological processes, we calculated the conditional probabilities of a metabolite being significantly altered in both genotypes. The likelihood of the observed overlaps occurring by chance was found to be 4.0×10^−7^%, 4.1×10^−9^%, 0.006% and 0.02% for the brain, gut, muscle and whole flies, respectively.

**Fig. 6.**
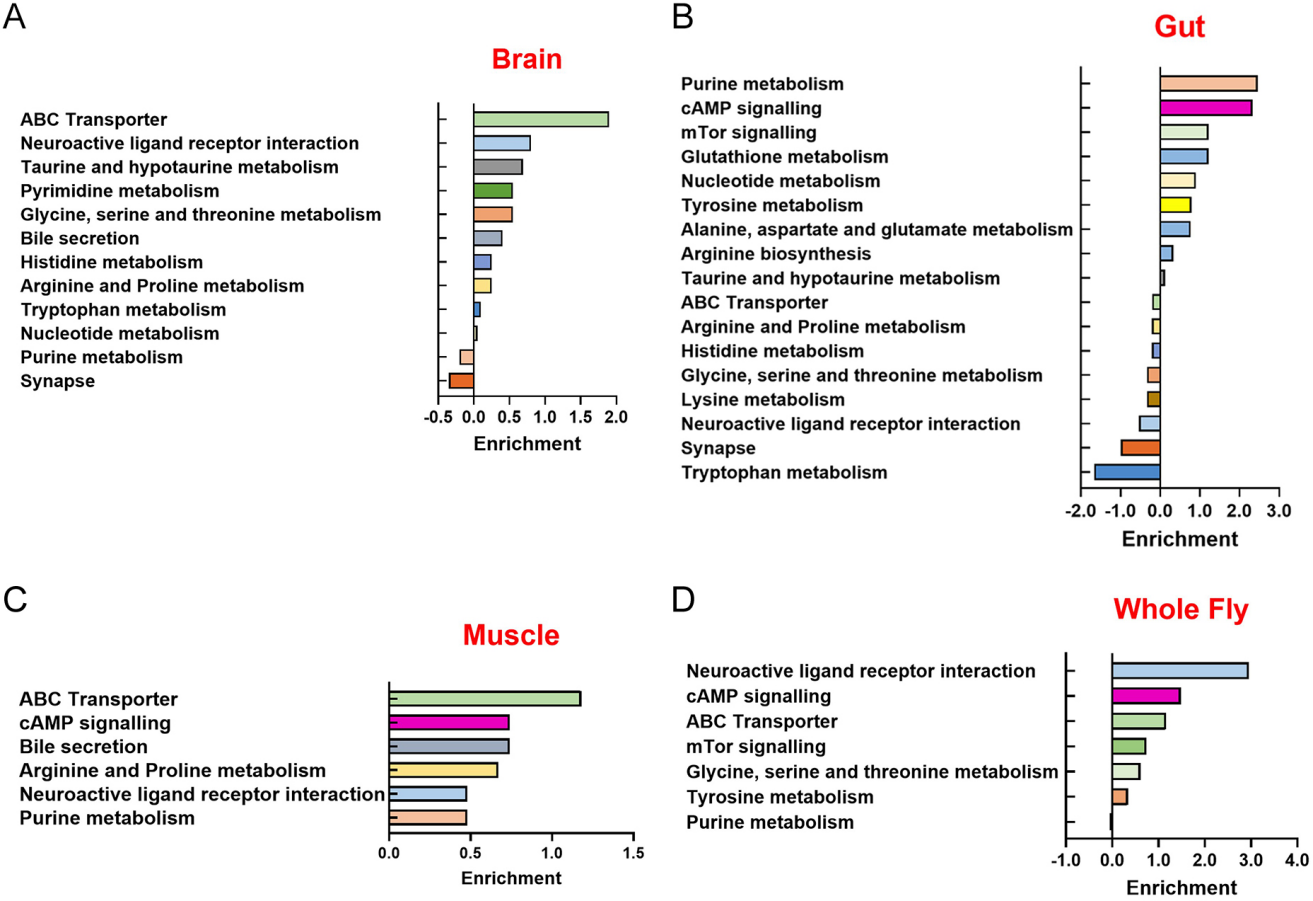
**The ABC transporter, cAMP signalling, purine metabolism and neuroactive ligand-receptor interaction are altered by DA in both PLAN models.** (A-D) Enrichment is the total number of significant metabolites per pathway, correcting for pathway size. DA treatment and ageing in brain, gut, muscle and whole flies. Metabolic pathways that are altered by DA in the brain (A), gut (B), muscle (C) and the whole fly (D) are found in both PLAN models.

Given this, we next examined the metabolic pathways affected by DA, prioritising pathway enrichment over the total number of altered metabolites, as pathway sizes vary. In the brain and, to a lesser degree, in the muscle and whole-fly samples, metabolites associated with the ATP-binding cassette (ABC) transporter pathway were significantly enriched (Fig. 6A,C). In the gut, significant changes were seen in purine metabolism and metabolites associated with cAMP signalling (Fig. 6B). In whole flies, in addition to the ABC transporter family, we observed alterations in metabolites associated with the neuroactive ligand-receptor interaction pathway (Fig. 6D).

## DISCUSSION

### DA enhances lifespan and bang sensitivity in *Drosophila* PLAN models

Previous studies have suggested the potential therapeutic benefits of DA in various neurological disorders, including PLAN. As a key component of the medium-chain triglyceride (MCT) ketogenic diet, DA and other MCFAs have been explored for their role in treating conditions like epilepsy due to their ability to readily cross the blood–brain barrier (Ebert et al., 2003; Johnson et al., 1990; Spector, 1988). In neurodegenerative diseases like PLAN, neuronal energy metabolism is compromised (Maalouf et al., 2009; Cunnane et al., 2016; Augustin et al., 2018; Stafstrom and Rho, 2012). DA has also demonstrated neuroprotective properties through multiple mechanisms, including the reduction of inflammation, which is a major contributor to neurodegenerative disease progression (Huang et al., 2014a; Glass et al., 2010; Frank-Cannon et al., 2009). Impaired mitochondrial function is another vital factor in neurodegenerative diseases (Trushina and McMurray, 2007; Golpich et al., 2017; Beal, 1998), and DA has been shown to increase mitochondrial complex I activity and mitochondrial count (Hughes et al., 2014; Pain et al., 2024; Malapaka et al., 2012). Lastly, MCFAs are known to stimulate the production of brain-derived neurotrophic factor (BDNF), which promotes neuronal health and resilience in neurodegenerative conditions (Sharma et al., 2023; Miranda et al., 2019; Gao et al., 2022). Given these potential benefits, this study focused on investigating the effects of DA in *Drosophila* models of PLAN, assessing its impact on lifespan, healthspan, and underlying molecular and cellular mechanisms.

Male flies in both PLAN models also exhibited shortened lifespans and impaired locomotion (Kinghorn et al., 2015; Lin et al., 2018). While studying the effects of DA in both sexes is important, our study focused on female flies for several reasons. Male flies tend to engage in more frequent and intense aggressive interactions (Monyak et al., 2021), which may increase variability in lifespan due to higher extrinsic mortality. Additionally, males generally exhibit higher baseline activity levels (Watanabe et al., 2020), potentially leading to greater variability in energy expenditure, and introducing additional noise in locomotion and stress resistance assays.

The *iPLA-VIA^Δ174^* flies displayed a greater reduction in median lifespan than *iPLA-VIA^EY05103^* flies, as indicated by our experiments (Fig. 1C,D) and previous literature (Kinghorn et al., 2015; Lin et al., 2018). The *iPLA-VIA^EY05103^* line was created by P-element insertion in the 5′ regulatory region of the *iPLA-VIA* gene, disrupting gene expression (Kinghorn et al., 2015). In contrast, the *iPLA-VIA^Δ174^* line was generated through the imprecise excision of the P-element, resulting in a 1335 bp deletion, and complete loss of gene expression and function (Lin et al., 2018). This complete loss likely contributed to the greater lifespan reduction in *iPLA-VIA^Δ174^* flies. Our experiments demonstrated a significant lifespan extension in both PLAN models following DA treatment (Fig. 1C,D). However, the absence of improvements in healthspan (Fig. S3A-D) suggests that DA does not suppress the age-associated decline in physical function in the *Drosophila* PLAN models.

Although DA showed no effect on climbing and locomotion, it did reduce bang sensitivity in *iPLA-VIA^Δ174^* flies (Fig. 2A,B). The bang-sensitivity test, which evaluates *Drosophila* mutants susceptible to mechanical shocks, measures hyperactivity and paralysis after a shock. Many genes implicated in bang sensitivity are associated with mitochondrial function, as supported by previous studies showing mitochondrial dysfunction in bang-sensitive mutants (Sen and Cox, 2017; Pareek et al., 2018; George et al., 2019).

Prior research by Mori (Mori et al., 2019) found that *Drosophila iPLA-VIA* null mutants exhibit a higher proportion of shorter phospholipid acyl chains. These chains alter membrane properties and increase curvature, leading to defects in synaptic vesicle dynamics, ER stress and neurodegeneration. Dietary supplementation of linoleic acid, a polyunsaturated fatty acid with 18 carbons, has been shown to enhance membrane fluidity and restore phospholipid composition (Yehuda et al., 2002). This is possibly because its unsaturated bonds confer greater flexibility and enable it to serve as a precursor for bioactive lipids involved in signalling (Ferreri et al., 2020). In contrast, fully saturated stearic and myristic acids do not provide the same benefit, as they contribute to more rigid membranes and are primarily used for structural roles or energy storage, rather than for phospholipid remodelling (Catalá, 2015). Similarly, DA is less suitable for incorporation into membrane phospholipids due to its fully saturated structure and relatively short chain length, and is more likely to exert its effects through other mechanisms.

### DA treatment improves resistance to heat-shock stress but not locomotor function

Previous studies have shown that certain pharmaceutical compounds that promote longevity also improve resistance to various stressors (Lashmanova et al., 2015; Schriner et al., 2013; Strilbytska et al., 2020). Our results showed that a low concentration of DA improved heat-shock stress resistance in young flies (Fig. 2C), consistent with the finding that dietary components can influence stress resistance in *Drosophila* (Strilbytska et al., 2021). Additionally, transcriptional analysis revealed an increased *Atg1* expression in PLAN model flies following DA treatment (Fig. 3E). The observed improvement in heat-shock stress resistance and increased *Atg1* expression suggested potential mechanisms through which DA may influence lifespan.

Atg1 plays an important role in the initiation of autophagy by regulating the formation of autophagosomes (Mizushima, 2010). Increased *Atg1* expression has been linked to lifespan extension in *Drosophila* and a reduction in oxidative stress (Simonsen et al., 2008), with long-lived *Atg1* over-expressing flies exhibiting enhanced resistance to heat-shock stress (Bjedov et al., 2020). Increased *Atg1* expression also boosts mitophagy, helping to clear dysfunctional mitochondria and reduce reactive oxygen species (ROS), which improves resistance to thermal stress (Bjedov et al., 2020; Liu and Lu, 2010; Ma et al., 2018). A possible explanation for our findings is that DA-induced upregulation of *Atg1* may enhance both autophagy and mitophagy, contributing to the greater heat-shock resistance detected in *iPLA-VIA^Δ174^* flies treated with DA. Interestingly, DA treatment specifically downregulated *Atg8a* expression in *iPLA-VIA^Δ174^* flies at day 27, while having no effect in younger flies (Fig. S5A). *Atg8a* is a key modulator of autophagy involved in the expansion and closure of the autophagosome. It has been used as a marker for autophagic flux in *Drosophila* (Mizushima, 2020; Kapahi et al., 2004). Although it may seem contradictory to the increased *Atg1* expression, these two autophagy-related proteins are involved in different steps of the autophagy process. Elevated *Atg1* expression and reduced *Atg8a* expression represent an increase in autophagy initiation with a lowered rate. Moreover, this late-life reduction in *Atg8a* expression does not necessarily contradict the positive effect of DA on longevity. One possible explanation is that DA enhances protective signalling pathways in early adulthood, promoting longevity, while later suppressing autophagy to prevent detrimental overactivation in older flies (van Heemst, 2010). Alternatively, DA could boost other cellular maintenance processes such as proteostasis, antioxidant defences or mitochondrial function, reducing the need for high autophagic activity later in life. Additionally, autophagy may only need to stay above a crucial threshold to maintain cellular homeostasis, meaning a moderate reduction in *Atg8a* expression could still preserve sufficient autophagic function while avoiding excessive autophagy.

Our data further revealed the ability of DA to modulate key pathways involved in stress resistance and cellular homeostasis, highlighting its potential as a therapeutic candidate for conditions associated with mitochondrial dysfunction. However, the lack of a significant impact of DA on starvation and oxidative stress resistance (Fig. S3E,F) indicates that the effect of DA may be limited to specific stress pathways. Similarly, antioxidant enzyme activity assays showed that DA had no significant impact on catalase or superoxide dismutase (SOD) activity in *iPLA-VIA^Δ174^* flies (Fig. S3G,H). The absence of improvement in starvation, oxidative stress resistance and antioxidant enzyme activity suggest that while DA treatment promotes longevity, it does not significantly alter metabolic pathways related to energy storage or antioxidant defence.

### DA modulates IIS/TOR and sirtuin pathways, protein homeostasis and autophagy

Gene expression analysis provides insights into the potential molecular mechanisms by which DA extends lifespan. In DA-treated *iPLA-VIA^Δ174^* flies, *InR* and *4E-BP* expression increased (Fig. 3A,B), while *foxo* levels remained unchanged (Fig. S4a). Since the insulin/TOR pathways are crucial regulators of lifespan and metabolic homeostasis, the modulation of these pathways by DA may underlie the observed lifespan extension (Teleman, 2009). The lack of a significant age-related change in *InR* expression suggests that the transcriptional shift is biologically modest (Fig. 3A). This is counterintuitive, given that IIS is associated with lifespan extension in *Drosophila* and other model organisms (Kang et al., 2017; Kasai et al., 2019). A slight increase in *InR* expression with age may therefore not indicate a meaningful change in IIS pathway activity. Expression data alone may not capture the full functional output of IIS, which is regulated post-transcriptionally through mechanisms such as receptor phosphorylation, feedback loops, and the activity of downstream effectors like Akt and FOXO (Hwangbo et al., 2004; Vasudevan et al., 2017). For example, FOXO localisation and activity are tightly regulated independently of *InR* mRNA levels and are stronger indicators of IIS function (Giannakou et al., 2004). It is also possible that *InR* upregulation is compensatory, reflecting a feedback response to declining IIS activity rather than causing it (Hay and Sonenberg, 2004). Thus, observed longevity phenotypes may occur despite or independently of these modest transcriptional changes.

In *Drosophila*, the IIS and TOR pathways interact to form the IIS/TOR signalling network (Carvalho et al., 2017). Elevated *InR* expression may lead to decreased 4E-BP activity (Carvalho et al., 2017), suggesting that the increased 4E-BP expression could either compensate for higher *InR* levels or be a result of DA independently influencing *4E-BP* expression. One possible pathway involved is the GCN2-ATF4 signalling axis, which is activated in response to amino acid restriction, mitochondrial dysfunction and bacterial infection, and has been shown to induce 4E-BP expression (Balaji et al., 2018; Zang et al., 2024; Damschroder et al., 2022). Interestingly, DA enhanced *4E-BP* expression on days 5 and 27, but reduced it on day 20 (Fig. 3B). These fluctuations may reflect age-related regulatory dynamics, as *4E-BP* is modulated by both the insulin and mTOR pathways. Additionally, accumulating oxidative stress and mitochondrial dysfunction could modulate *4E-BP* expression as part of an adaptive response to ageing. The activation of 4E-BP relies on its phosphorylation status (Bettencourt et al., 2008). When unphosphorylated, 4E-BP binds to eIF4E and suppresses cap-dependent translation (Dunn et al., 2023b). DA might influence this regulatory process by modulating the activity of kinases or phosphatases that control the phosphorylation of 4E-BP, thereby indirectly affecting its activity.

DA treatment did not affect *foxo* expression levels (Fig. S4A). However, since the activity of FOXO is primarily regulated post-transcriptionally through IIS-mediated phosphorylation (Scott et al., 2004; Warren et al., 2020), further studies are needed to determine whether some of the effects of DA are mediated via this transcription factor.

Overexpression of *Sirt1* extends lifespan (Frankel et al., 2011), while its downregulation shortens it (Aström et al., 2003). Previously, DA has been shown to activate mammalian SIRT1 and increase expression of *Sirt3* in mouse neurons (Youle and Narendra, 2011). This aligns with our findings that DA elevates *Sirt1* expression in *iPLA-VIA^Δ174^* flies (Fig. 3C). DA treatment also upregulated *Hsp70* expression in PLAN flies across all time points (Fig. 3D). Hsp70 is a molecular chaperone that plays a key role in stress resistance by promoting the proper folding of damaged proteins (Feder and Hofmann, 1999). Its rapid upregulation in response to environmental stresses is linked to increased thermal resistance (Metaxakis et al., 2018), providing a plausible explanation for the positive effect of DA on heat tolerance in PLAN flies.

Medium-chain fatty acids like DA are known to promote autophagy, offering potential therapeutic benefits for neurodegenerative diseases (Kanabus et al., 2016). The increased *Atg1* expression in DA-treated flies (Fig. 3E) suggests that DA treatment may promote longevity by enhancing autophagy initiation, as elevated *Atg1* expression in flies has been linked to lifespan extension and increased heat-shock resistance (Bjedov et al., 2020). Since Atg1 is negatively regulated by mTORC1 (Hughes et al., 2014), and DA suppresses mTORC1 activity (Chen et al., 2021), DA may upregulate *Atg1* through mTORC1 inhibition. Atg1 also promotes mitophagy, a cellular process that enhances stress resistance and longevity by clearing dysfunctional mitochondria and preventing the accumulation of reactive oxygen species (ROS) (Olvera-Rosales et al., 2021). This mechanism may further contribute to the lifespan-extending effects of DA.

Although DA did not significantly alter mitochondrial gene expression, its known effect on mitochondrial function (Han et al., 2022) suggests that it may enhance mitochondrial activity through non-transcriptional mechanisms, such as regulation of enzyme activity (Rees et al., 2009). Collectively, these findings suggest that DA influences key genes in the IIS/TOR, sirtuin, heat-shock stress response and autophagy pathways, which likely contribute to the enhanced stress resistance and lifespan extension observed in *iPLA-VIA^Δ174^* flies.

### DA induces metabolic shift in *Drosophila* PLAN flies

To investigate the molecular mechanisms underlying the physiological effects of DA, we performed metabolomic analysis on *Drosophila* PLAN models following DA treatment. Four metabolic pathways – ABC transporters, purine metabolism, cAMP signalling and neuroactive ligand receptor interaction – were identified as key effectors of DA function, though significant tissue-specific differences were observed (Fig. 4).

Our data indicate significant metabolic alterations in the gut of both PLAN models upon DA treatment. The strong conservation of age- and treatment-related metabolic shifts in the gut suggests that this tissue plays a crucial role in the ability of DA to mitigate disease-associated phenotypes. The presence of the microbiome makes the gut a highly metabolically active tissue and key to regulating many processes. The gut microbiome is a complex community of microorganisms residing in the gastrointestinal tract that has been recognised for its role in human health and disease (Jha et al., 2019). In recent years, there has been a growing acknowledgement of the importance of the gut–brain axis in neurodegeneration. Alterations in the composition of gut microbiota have been linked to various conditions, including neurodegenerative diseases, suggesting a potential microbiota–intestine–brain axis (Huang et al., 2014b). Notably, emerging studies suggest bidirectional communication between the gut microbiota and the brain, potentially influencing pathways involved in the pathogenesis of neurodegenerative diseases (Abuznait and Kaddoumi, 2012). These findings underscore the role of the gut as a potential mediator of the protective effects of DA in *Drosophila* PLAN models.

### DA modulates ABC transporters, purine metabolism, cAMP signalling and neuroactive ligand–receptor interactions

ATP-binding cassette (ABC) transporters form a large family of proteins that facilitate the translocation of diverse substrates across cellular membranes (Castelli et al., 2019). They are essential for maintaining cellular homeostasis, particularly through lipid transport and the efflux of xenobiotics and drugs (Klucken et al., 2000). In *Drosophila*, overexpression of the ABC transporter *Mrp4* increases resistance to oxidative stress and extends lifespan, while its loss shortens lifespan (Tarling et al., 2013). Their role in neurodegenerative diseases, such as Alzheimer's, includes regulating lipid homeostasis, a crucial factor for neuronal function (Cicero et al., 2023). Disruption of lipid metabolism is a hallmark of many neurodegenerative conditions, including PLAN (Huang et al., 2021). Since ABC transporters actively mediate phospholipid translocation (Musselman and Kühnlein, 2018; Yan et al., 2016), loss of *iPLA-VIA* function likely impairs phospholipid metabolism, affecting the intracellular distribution of other metabolites. Our metabolomics data reveal modulation of the ABC transporter pathway in DA-treated whole flies (Fig. 6D), which also show upregulation of *Hsp70* and *Atg1* (Fig. 3D,E), markers of enhanced proteostasis and autophagy initiation (Evans et al., 2010; Bjedov et al., 2020). These findings suggest that DA treatment supports cellular clearance mechanisms by modulating ABC transporter activity, promoting protein chaperone expression and initiating autophagy. Together, these effects help remove cellular waste, maintain protein homeostasis and potentially contribute to lifespan extension.

Purine metabolism is another pathway affected by DA treatment. Purines play crucial roles in various cellular processes, including DNA synthesis and cell proliferation, energy transfer, and neurotransmission (Lee, 2015); disruptions in purine metabolism can lead to cellular energy deficits, increased oxidative stress and shortened lifespan in *Drosophila* (Frappaolo and Giansanti, 2023; Gerhart-Hines et al., 2011). The alterations in adenosine levels upon DA treatment (Pain et al., 2024) indicate that DA may be modulating purine metabolism, potentially offering therapeutic benefits by restoring metabolic balance and reducing oxidative stress, likely contributing to the lifespan extension observed in PLAN flies treated with DA.

The cyclic AMP (cAMP) signalling pathway plays a crucial role in regulating gene expression, cell growth and differentiation (Zhang et al., 2022), and appears to be influenced by DA. In *Drosophila*, cAMP signalling is essential for synaptic plasticity and memory formation (Lee, 2015), with mutations in core components such as adenylyl cyclase and phosphodiesterase linked to learning impairments (Lee, 2015). Given its importance in neural regulation, disruptions in cAMP signalling may contribute to PLAN pathogenesis, while the influence of DA on this pathway could offer neuroprotective effects. Notably, DA treatment elevates *Sirt1* expression (Fig. 3C), aligning with reports that cAMP signalling enhances SIRT1 activity (Gerhart-Hines et al., 2011). Our metabolomics data further support this, showing that DA modulates the cAMP signalling in whole flies (Fig. 6D). In *Drosophila*, exogenous cAMP has been shown to extend lifespan by boosting Sirtuin protein levels and activity (Wang et al., 2015). Mechanistically, cAMP activates calmodulin kinase kinase II (CaMKKII), which in turn increases AMPK phosphorylation, promotes NAD^+^ production and activates *Sirt1* (Park et al., 2012). The observed upregulation of *Sirt1* alongside cAMP pathway modulation suggests that DA may promote longevity through the cAMP–CaMKKII–AMPK–Sirt1 signalling axis.

Lastly, the neuroactive ligand–receptor interaction pathway, which is fundamental for neurotransmission, may also be influenced by DA. By modulating this pathway, DA could enhance neuronal communication, potentially mitigating cognitive and motor impairments associated with PLAN and other neurodegenerative disorders.

In conclusion, our study demonstrates diverse effects of DA in *Drosophila* PLAN models, revealing its potential therapeutic relevance. While the exact molecular mechanisms remain to be fully elucidated, modulation of key metabolic pathways by DA lays the foundation for further research into its application as a therapeutic intervention for PLAN and related neurodegenerative diseases.

## MATERIALS AND METHODS

### Fly stocks

The wild-type control *white^Dahomey^* (*w^Dah+^*) strain was initially created by backcrossing the *w1118* strain into the outbred wild-type Dahomey background (Broughton et al., 2005). The *w^Dah+^* will be referred to as WT. The *iPLA-VIA^EY05103^* strain was created by introducing a P-element (P[EPgy2]) into the 5′ UTR of the *iPLA2-VIA* gene (P[EPgy2] iPLA2-VIA^EY05103^), resulting in a significantly reduced *iPLA2-VIA* gene expression (Kinghorn et al., 2015; Iliadi et al., 2018; Banerjee et al., 2021). The *w^Dah+^* and *iPLA-VIA^EY05103^* strains were a gift from Kerri Kinghorn, University College London, UK (Kinghorn et al., 2015). The *iPLA-VIA^Δ174^* line was created by imprecise excision of the P-element of the *iPLA2-VIA^EY05103^* fly, resulting in the deletion of 1335 bp and the lack of detectable iPLA-VIA protein by Western blotting (Lin et al., 2018). The *iPLA-VIA^Δ174^* line was obtained from Bloomington Drosophila Stock Center (80311). The expression of the *iPLA2-VIA* gene in the *iPLA-VIA^Δ174^* flies was validated by qPCR analysis using *iPLA2-VIA*-specific primers (Table S1). The data were analysed by an unpaired, two-tailed *t*-test using GraphPad Prism.

### Fly husbandry

All flies were kept on 1×SYA fly food consisting of 1.5% agar, 10% sugar, 10% yeast, 0.3% propionic acid and 0.3% nipagin. All flies were maintained at 25°C and 60% humidity under a 12:12 h dark: light cycle.

### Embryo collection

Fly cages with egg collection medium were maintained at 25°C for a maximum of 24 h. Eggs were then collected and washed with 1×phosphate-buffered saline (PBS) before transferring 20 μl of egg/PBS suspension into the 1×SYA bottles. Bottles were stored at 25°C until all flies emerged; the flies that emerged were kept in the bottles for 2 extra days to mate. The ‘twice-mated’ flies were used in all experiments.

### Compound administration

DA (MW=172.26 g/mol) was dissolved in DMSO and added to standard fly food to create final concentrations of 50 μM, 250 μM and 1 mM DA. Control food was prepared by adding the same volume of DMSO. Twice-mated flies were transferred onto DMSO- or compound-containing food 2 days after eclosion. The flies were continuously fed compound- and DMSO-containing food throughout their lifespan.

### qPCR

Total RNA was extracted using the TRIzol reagent method (Green and Sambrook, 2020). Flies (females) were maintained under the same conditions as those used for the lifespan assays. RNA was reverse transcribed with the UltraScript cDNA Synthesis Kit (PCR BioSystems) according to the manufacturer's protocol. qPCR was performed on cDNA using the GoTaq qPCR System (Promega) and Rotor-Gene Q (Qiagen). qPCR of *iPLA2-VIA* mutants (Fig. 1) was carried out in triplicate on cDNA from a single RNA extraction from 12 flies (three technical replicates). All other gene expression analyses (Fig. 3, Figs S4 and S5) used cDNA from three independent RNA extractions from four flies each (12 in total), yielding three biological replicates. Relative quantification was performed by normalising target gene C_t_ values to *Drosophila β-tubulin*. *β-tubulin* C_t_ values from multiple qPCR runs were compared by two-way ANOVA with Tukey's multiple comparison test (GraphPad Prism), confirming that *β-tubulin* expression was unaffected by age or DA treatment (Fig. S1). Primer sequences are listed in Table S1. Ct values were analysed using the ΔΔCt method to calculate relative quantification (RQ). Statistical analyses of RQ values were conducted by two-way ANOVA with Tukey's multiple comparison test in GraphPad Prism.

### Lifespan assay

150 female flies were collected for each genotype and treatment (10 vials×15 flies). Every 2 to 3 days, flies were transferred onto fresh food, and the numbers of dead and censored flies were recorded. The results were analysed using the lifespan scoring form from PiperLab (Piper et al., 2014). The median lifespan is described as the age when the survival curve drops to 50%. The maximum lifespan is defined as the median lifespan of the longest-lived 10% of individuals. The significant difference is evaluated after the experiments using the log-rank test with chi-squared *P*-value on survivorship data.

### Negative geotaxis (climbing) assay

A modified version of the previously published climbing protocol (Woodling et al., 2020) was used. 75 female flies were collected for each genotype and treatment (5 vials×15 flies). Flies were kept under the same conditions as the flies for lifespan assays and transferred out for climbing assay every 7 days. The assay was performed by transferring the flies into a 20 cm vertical column and left at room temperature for 20 min to acclimate. After resting, flies were tapped four times to the bottom of the vials and allowed to climb upwards. An Apple iPod Touch camera was used to record 25 s videos starting from the initial tap. Then, an image from the recording was captured 12 s after the last tap for *iPLA-VIA^EY05103^* flies. The image for *iPLA-VIA^Δ174^* flies was captured 20 s after the last tap due to their smaller body size and more-severe climbing phenotype. The height of each fly was analysed by first calibrating the height in pixels to the height in cm using a scale placed next to the flipper when recording, followed by manual selection in ImageJ software (Schneider et al., 2012). Finally, the results were analysed by linear regression analysis and two-way ANOVA with Tukey's multiple comparison tests using GraphPad Prism.

### Locomotion assay

The locomotion assay was modified from a previously published protocol (Barwell et al., 2021). 75 female flies were collected for each genotype and treatment and kept under the same conditions as the flies for lifespan assays. Every 7 days, 25 flies were selected randomly for video recording. Flies were transferred into circular arenas with a diameter of 2.5 cm. Once all arenas were loaded, the base and the chamber strip were placed on a light pad. An Apple iPod was clamped 40 cm above the light pad to capture a video containing all 25 chambers. Videos were captured at 25°C with the light pad on. Flies were allowed to acclimatise to the chamber for 1 min after loading, and then the flies were recorded for 3 min at 30 frames per second. Each treatment and genotype were measured in triplicate. The movement of flies was analysed by the video tracking software EasyFlyTracker (Qu et al., 2022) (http://easyflytracker.cibr.ac.cn/#/document). The software required a Python (version 3) environment and the installation of scikit-learn software (Pedregosa et al., 2011). Statistical analyses were performed using linear regression analysis and two-way ANOVA, followed by Tukey's multiple comparison tests.

### Bang-sensitivity test

The bang-sensitivity test was performed following the protocol from Lin et al. (2018). Female flies were maintained under the same conditions as those used for the lifespan assay. The experiment was carried out on days 8 and 15 post-eclosion. For each time point, five vials, each containing 10-15 flies, were vortexed at maximum speed for 15 s. Recovery time, defined as the time taken for each fly to return to an upright position, was recorded. Statistical analyses were conducted in GraphPad Prism using two-way ANOVA with Tukey's multiple comparison tests and linear regression.

### Heat-shock stress assay

The heat-shock stress assay was conducted on both young (day 3) and old flies (day 24) as previously described (Lee et al., 2019). Flies were kept under the same conditions as those used for lifespan assays. At each time point, at least 60 female flies were transferred into new vials and placed in 39°C incubators. Every 10 min, the number of dead flies was recorded. Significant differences were evaluated using the log-rank test with chi-squared *P*-values based on survivorship data.

### Starvation assay

The starvation stress was performed as described by Lee et al. (2019). 150 female flies treated with DA for 7 days were transferred to vials containing 0.8% agar. Every 12 h, the flies were tipped into the new vial with 0.8% agar, and dead flies were recorded after each transfer. Significant differences were evaluated using the log-rank test with chi-squared *P*-values based on survivorship data.

### Oxidative stress assay

The oxidative stress assay was conducted following the protocol outlined by Lee et al. (2019). After 7 days of DA treatment, 150 female flies were transferred to vials containing 15 mM methyl viologen dichloride hydrate (Sigma-Aldrich) dissolved in 5% sucrose. Methyl viologen dichloride hydrate, also known as paraquat, is a widely studied herbicide that induces oxidative stress by disrupting mitochondrial function. Paraquat interferes with mitochondrial electron transport by accepting electrons from complex I and transferring them to molecular oxygen, generating reactive oxygen species (ROS) (Cochemé and Murphy, 2008). The excessive ROS production can cause oxidative damage to lipids, proteins and DNA (Juan et al., 2021). Exposure to paraquat in *Drosophila* has been shown to disrupt mitochondrial function and increase oxidative stress (Hosamani and Muralidhara, 2013). A layer of filter paper saturated with 100 μl of the 15 mM methyl viologen dichloride hydrate was placed at the bottom of vials containing of 0.8% agar. Every 12 h, the flies were transferred to fresh food containing the methyl viologen dichloride hydrate solution, and the dead flies were recorder at each transfer. Significant differences were evaluated using the log-rank test with chi-squared *P*-values based on survivorship data.

### Antioxidant enzyme activity assay

Thirty female flies treated with DA for 7 days were collected for the assays (Lee et al., 2019). The activities of superoxide dismutase (SOD) and catalase (Cat) were determined using the SOD Assay Kit-WST (19160, Sigma-Aldrich) and the Catalase Activity Assay Kit (Colorimetric/Fluorometric) (ab83464, Abcam), following the protocols provided by the respective manufacturers. The statistical significance was determined by an unpaired, two-tailed *t*-test.

### Metabolomics

#### Solvents and reagents

All solvents and chemicals used, including methanol, water, acetonitrile, ammonium formate and methyl-tertiary butyl ether (MTBE), were LC-MS grade and sourced from Fisher Scientific or Sigma-Aldrich. Internal standards used were tripentadecanoin for the organic phase and L-valine 13C515N (95%) for the aqueous phase, both obtained from Sigma-Aldrich.

#### Metabolite extraction

Metabolomic samples were collected at four-time points. The first time point was on day 7 post-eclosion. The second time point was determined based on the age at which the survival curves of the control and treatment groups began to diverge. The third and fourth time points were collected 5 and 10 days after the second time point, respectively. Female flies treated with DA were maintained under the same conditions as described in the lifespan assay before being collected for metabolomic analysis. At each time point, 20 flies were collected, with 15 of them dissected to obtain tissue samples, including the brain, muscle and mid-gut. All dissection were conducted in 1×PBS. The brain, muscle and mid-gut were dissected following the protocol from Tito and Xiao (Tito et al., 2016; Xiao et al., 2017). The tissues were maintained in 30 μl of HPLC-grade methanol after dissection. Five flies were collected as whole-fly samples at each time point. Tissues were pooled from 15 flies into three independent biological samples (five flies per pool) to provide sufficient material for metabolomic profiling. Each pool was processed independently and measured in triplicate injections (technical replicates). All sample preparation and LC-MS runs were randomised and interleaved with pooled quality-control (QC) injections. Throughout the manuscript, *n* refers to the number of independent biological samples measured.

Metabolite extraction was carried out using a modified version of a previously published method (Ebshiana et al., 2015). A 4 mm steel ball bearing and 30 µl of methanol were added to each sample tube, which was then vortexed for 5 min for mechanical homogenization. To each homogenate, the following reagents were added in sequence, 5 µl of aqueous phase internal standard solution (2.5 mM L-valine 13C515N in 80:20 methanol:water), 140 µl of MTBE with 15 µM tripentadecanoin (organic phase internal standard) and 10 µl of methanol. The samples were then incubated at 4°C for 10 min to disrupt cell membranes. After incubation, samples were transferred to HPLC vials with 250 µl glass inserts, followed by the addition of 40 µl of 0.15 mM ammonium formate in water. The samples were centrifuged at 5000 ***g*** for 5 min, and 30 µl of organic and 30 µl of aqueous phase were retained for analysis. A pooled quality control (QC) sample was created by combining 5 µl from all analytical samples, and extraction blanks were prepared by replacing larval tissue with 15 µl of HPLC-grade water and following the same extraction protocol.

#### Hydrophilic liquid interaction chromatography (HILIC) analysis of the aqueous phase

Chromatographic separation of aqueous-phase metabolites was conducted using an Agilent Infinity HPLC system. Mobile phase A consisted of 10 mM ammonium formate in water; mobile phase B consisted of 2.5 mM ammonium formate in 90:10 acetonitrile:water. Separation was performed on an Agilent Poroshell HILIC-z column (2.1×150 mm, 4 μm), maintained at 30°C with a flow rate of 0.25 ml/min.

The gradient began with 95% mobile phase B for the first 5 min, followed by a linear decrease to 90% at 6 min and 75% at 15 min. The column was then cleaned for 3 min at 20% mobile phase B before re-equilibrating under initial conditions for 7 min. Mass spectrometry was performed using an Agilent 6550 ion funnel QToF (Agilent), with data collected across a 50-1000 m/z range. The drying gas flow was 15 l/min, nebulizer pressure was 40 psi, gas temperature was 200°C, sheath gas temperature was 300°C and sheath gas flow was 12 l/min.

#### Reverse-phase analysis of the non-aqueous phase

Organic-phase metabolites were separated using an Agilent Poroshell C18 column (2.1×150 mm, 2.7 μm) maintained at 55°C, with a solvent flow rate of 0.50 ml/min. Mobile phase A consisted of 10 mM ammonium formate in water; mobile phase B was 10 mM ammonium formate in a methanol:MTBE mixture (2:1 v/v).

The gradient began with 80% mobile phase B, increasing linearly to 93% by 13 min, 94% by 20 min and 96% by 24 min. The column was cleaned for 6 min at 100% mobile phase B before re-equilibration at initial conditions for 5 min. Mass spectrometry was performed using an Agilent 6550 ion funnel QToF, with data collected over a 50-1200 m/z range. The drying gas flow was 15 l/min, nebulizer pressure was 35 psi, gas temperature was 200°C, sheath gas temperature was 120°C and sheath gas flow was 10 l/min.

### Data processing and statistical analysis

ProteoWizard (Fernandes et al., 2023) was used to convert .d files to .mzXML format for data processing. The .mzXML files were analysed in R (v3.6.0) using the CAMERA package. Peak identification and integration were performed using the ‘centwave’ method, which can deconvolute closely eluting and slightly overlapping peaks (Chambers et al., 2012; Kuhl et al., 2012). Data filtering was applied to retain only features present in all samples of at least one group and with an abundance at least five times higher than in extraction blanks. Multivariate statistical analyses were conducted on the combined HILIC and RP datasets using SIMCA (v13.0.4). Prior to analysis, data were scaled to unit variance (UV) and log-transformed (base 10). Principal component analysis (PCA) was used to identify potential outliers and explore macro trends. Partial least squares discriminant analysis (PLS-DA) was applied for class-variable modelling, with model performance evaluated using cumulative correlation coefficients [R2X(cum)] and predictive performance assessed by sevenfold cross-validation [Q2(cum)]. Model significance was determined using cross-validated residual ANOVA (CV-ANOVA).

Normality of data was assessed using the Shapiro–Wilks test. Normally distributed data were analysed with unpaired, two-tailed *t*-tests, and skewed data were analysed using the Mann–Whitney test, all performed in R (v3.6.0). Initial metabolite annotation was performed using an in-house library of metabolite standards, based on matches in accurate mass, retention time and fragmentation spectra. When no matches were found, public spectral libraries, including HMDB (hmdb.ca) and METLIN (metlin.scripps.edu), were searched to identify putative annotations.

### Conditional probabilities

Having observed overlap in which metabolites were significantly (*P*<0.05) altered in both genotypes, we wanted to see if this overlap could be explained by chance or was the results of overlapping biology; this was assessed using conditional probability. To calculate the probability of one metabolite being significant in both genotypes by chance, the probability of it being significant in *iPLA-VIA^EY05103^* was multiplied by the probability that it is significant in *iPLA-VIA^Δ174^* given it is significant in *iPLA-VIA^EY05103^* (see equations below). Once the probability of one metabolite overlapping by chance was calculated, the likelihood of multiple metabolites overlapping was calculated using a simple multiple event calculation, e.g. if the probability that one metabolite overlaps by chance is 0.1 (10%) then the likelihood that ten metabolites overlap by chance is 0.1^10^ (0.00000001%).

p(*iPLA-VIA^EY05103^*)×p(*iPLA-VIA^EY05103^*/*iPLA-VIA^Δ174^*)=p(*iPLA-VIA^EY05103^* and *iPLA-VIA^Δ174^*);

p(*iPLA-VIA^EY05103^*)=probability of being significant in *iPLA-VIA^EY05103^*.

p(*iPLA-VIA^EY05103^*/*iPLA-VIA^Δ174^*)=probability of being significant in *iPLA-VIA^Δ174^* given that it is significant in *iPLA-VIA^EY05103^*;

p(*iPLA-VIA^EY05103^* and *iPLA-VIA^Δ174^*)=probability of being significant in *iPLA-VIA^EY05103^* and *iPLA-VIA^Δ174^* (Dunn et al., 2023a).

### Pathway enrichment

Enrichment analysis was performed by considering the total number of metabolites with abundances altered by DA treatment within each metabolic pathway (based on KEGG pathways). This approach accounts for differences in pathway size, as some pathways contain more metabolites than others. To control for pathway size, an enrichment score was calculated to quantify how many more (or fewer) metabolites there were with significantly (*P*<0.05) changed abundances in response to DA treatment than would be expected by chance. The number of metabolites expected by chance in any given pathway was calculated by dividing the number of significant metabolites in that pathway by the total number of significant metabolites identified in that tissue and multiplying it by the total number of metabolites in that pathway that were measured. Once this was carried out, the ‘expected’ number of significant metabolites was subtracted from the ‘observed’ number of significant metabolites to give the enrichment.

### Western blotting

Six whole flies were homogenised in 2×Laemmli SDS sample buffer (Thermo Scientific, J61337.AC) with 5% DL-dithothreitol solution (DTT, Sigma-Aldrich, 43816) using micropestles. Lysates were boiled for 5 min at 95°C, then centrifuged at 17,000 ***g*** for 3-5 min. Proteins were separated on 10% SDS-PAGE gels at 80 V for 1.5 h, using PageRuler Plus Prestained Protein Ladder (Thermo Fisher Scientific, 26619) as a migration marker. Proteins were transferred to nitrocellulose membranes (Amersham Protran Premium 0.45 μM NC, Cytiva, 10600003) at 10 V overnight (16 h) at 4°C. Membranes were left to air dry for ∼7 h, then blocked for 1 h at room temperature using Intercept Blocking Buffer (Li-Cor, 927-60001). Membranes were incubated overnight (16 h) in blocking buffer with the following primary antibodies: rabbit polyclonal anti-ref2 (Abcam, ab178440) at 1:1000, rabbit polyclonal anti-Atg8a (Merck Millipore, ABC974) at 1:1000 and mouse monoclonal anti-tubulin (Sigma-Aldrich, MAB3408) at 1:500. Membranes were washed in TBS-T [Tris-buffered saline: 250 mM Tris (pH 8), 1.4 M NaCl, 50 mM KCl, with 0.1% Tween 20] for 3×5 min then protected from light and incubated for 1 h at room temperature in blocking buffer with the following secondary antibodies: goat anti-mouse IRDye 800CW (Li-Cor, 926-32210) at 1:10,000 and goat anti-rabbit IRDye 680RD (Li-Cor, 926, 68071) at 1:10,000. Membranes were washed in TBS-T for 3×5 min, rinsed with TBS, then imaged using a Odyssey CLx Imager (Li-Cor).

Relative protein levels were quantified on Image Studio (Li-Cor), using tubulin expression levels for normalisation. GraphPad Prism 9 software was used for the statistical analyses of relative protein levels. Two-way ANOVA tests were used, followed by Tukey's post-hoc tests. In all cases, *P*<0.05 is considered statistically significant (*P<0.05, **P<0.01, ***P<0.001, ****P<0.0001; n.s., not significant). The *n* in the figure legend denotes the number of biological replicates.

## Supplementary Material

10.1242/dmm.052184_sup1 Supplementary information
