# Supplementary material for: Decanoic acid extends lifespan and modulates metabolism in models of PLA2G6-associated neurodegeneration
Source: Dis Model Mech. 2025 Dec 4;18(11):dmm052184. doi: 10.1242/dmm.052184 (PMC12714139; doi:10.1242/dmm.052184)
Supplement: Supplementary information [file dmm-18-052184-s1.pdf]

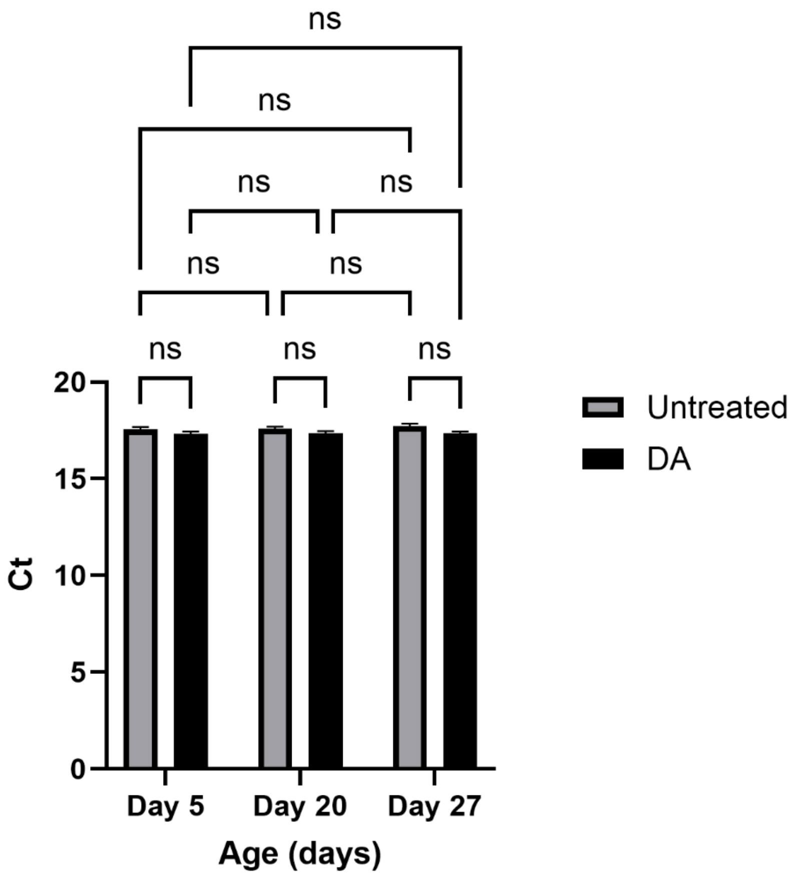

Fig. S1. DA treatment and age do not affect *β-Tubulin* expression. Ct values of *β-Tubulin* in *iPLA-VIA*$^{\Delta174}$ 5, 20, and 27 days post-eclosion showed no significant differences between DA-treated and untreated groups or across age groups. The interaction between treatment and age was not significant (p = 0.7469). Statistical analysis was performed using two-way ANOVA with Tukey's multiple comparisons in GraphPad Prism. n.s. = not significant (n = 42).

Disease Models & Mechanisms • Supplementary information

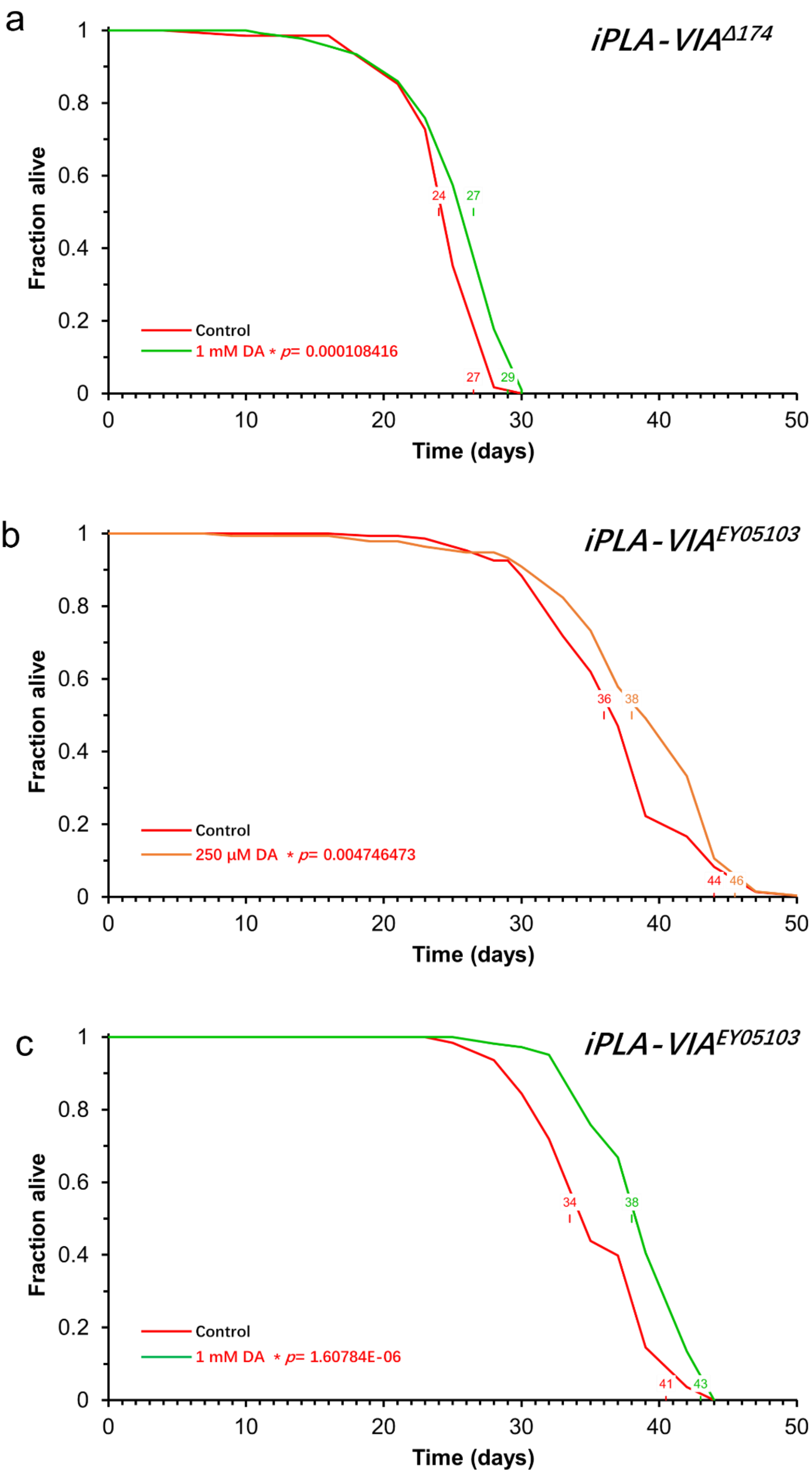
a
iPLA-VIA^Δ174
Fraction alive
Time (days)
Control
1 mM DA * p= 0.000108416
24
27
27
29
b
iPLA-VIA^EY05103
Fraction alive
Time (days)
Control
250 µM DA * p= 0.004746473
36
38
44
46
c
iPLA-VIA^EY05103
Fraction alive
Time (days)
Control
1 mM DA * p= 1.60784E-06
34
38
41
43

Fig. S2. DA extends lifespan in Drosophila PLAN model flies treated with DA (repeated experiments). a *iPLA-VIA*$^{\Delta 174}$ flies fed 1 mM of DA showed a significantly extended lifespan (n = 150). **b** and **c** DA extends lifespan of *iPLA-VIA*^{EY05103} flies at concentrations of 250 µM (b) and 1 mM of DA (c) (n = 150). All lifespan data were analysed using the log-rank test (* $p < 0.05$).

Disease Models & Mechanisms • Supplementary information

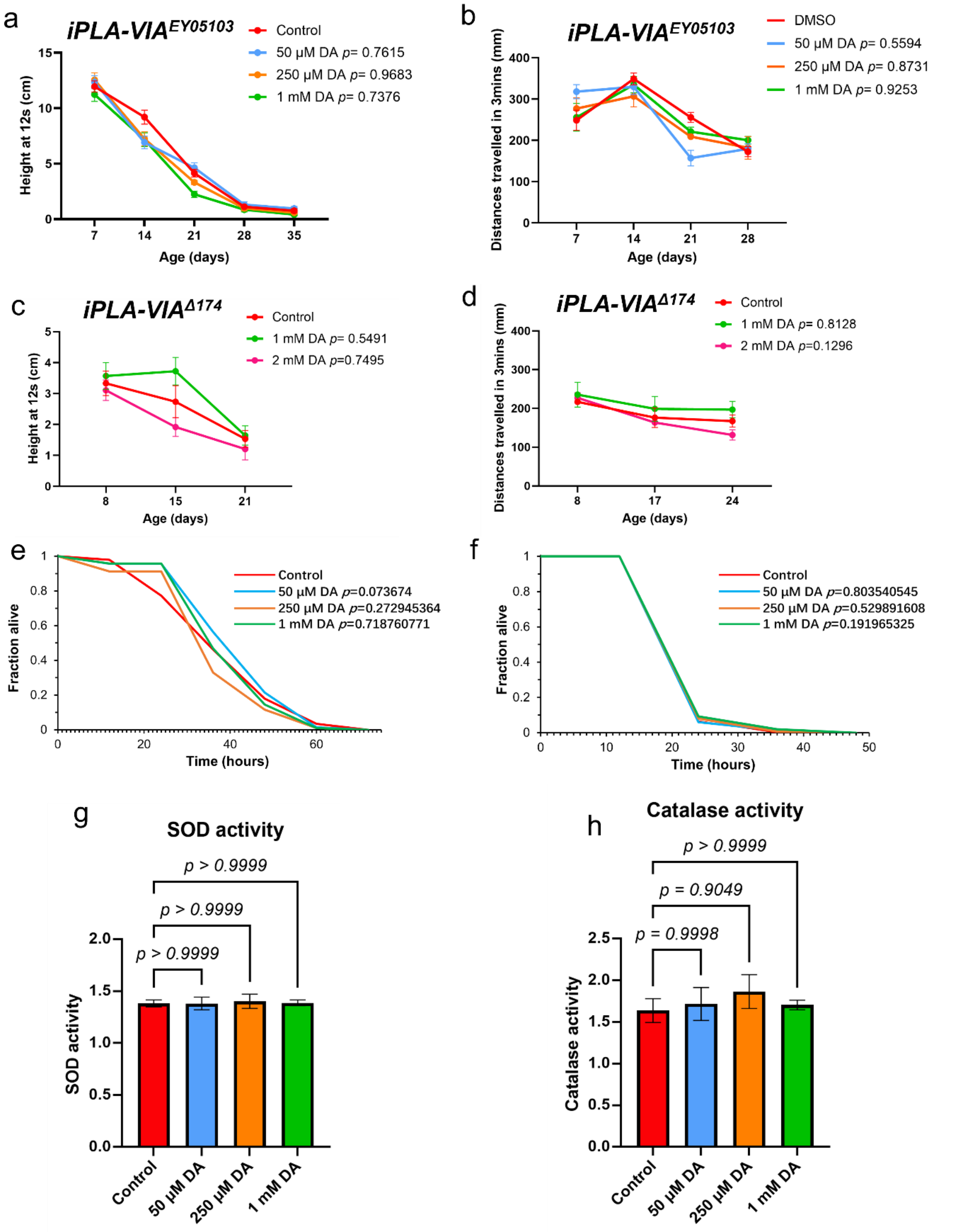

Fig. S3. DA does not affect climbing, locomotion, starvation stress, oxidative stress, and antioxidant enzyme activity in PLAN flies. a and **c** Climbing assays of *iPLA-VIA*^{EY05103} and *iPLA-VIA*$^{\Delta174}$ flies, respectively, fed different concentrations of DA (50 µM, 250 µM, 1 mM, and 2 mM). DA did not significantly improve climbing ability in any fly strain compared to DMSO controls (n = 75). **b** and **d** Locomotion assays of *iPLA-VIA*^{EY05103}, and *iPLA-VIA*$^{\Delta174}$

flies, respectively, treated with DA. DA at varying concentrations did not significantly affect the locomotion (n = 75). Statistical analyses for data from **a-d** were conducted using linear regression analysis and two-way ANOVA with Tukey's multiple comparisons tests. Error bars represent SEM, and differences were considered significant at $p < 0.05$. **e** Starvation stress assays. DA at varying concentrations did not significantly affect starvation stress resistance (n = 150). **f** Oxidative stress assay. DA had no significant effect on oxidative stress resistance (n = 150). Data for **e** and **f** were analysed using the log-rank test, with significance set at $p < 0.05$. **g** SOD (superoxide dismutase) assays of *iPLA-VIA*$^{\Delta 174}$ flies fed 50 μM, 250 μM and 1 mM of DA. No significant change in SOD activity upon DA treatment. **h** Catalase assays of *iPLA-VIA*$^{\Delta 174}$ flies showed no effect of DA on catalase activity (n = 30). Values from **g** and **h** were analysed using two-way ANOVA with Tukey's multiple comparisons tests in GraphPad Prism. Error bars represent SEM.

Disease Models & Mechanisms • Supplementary information

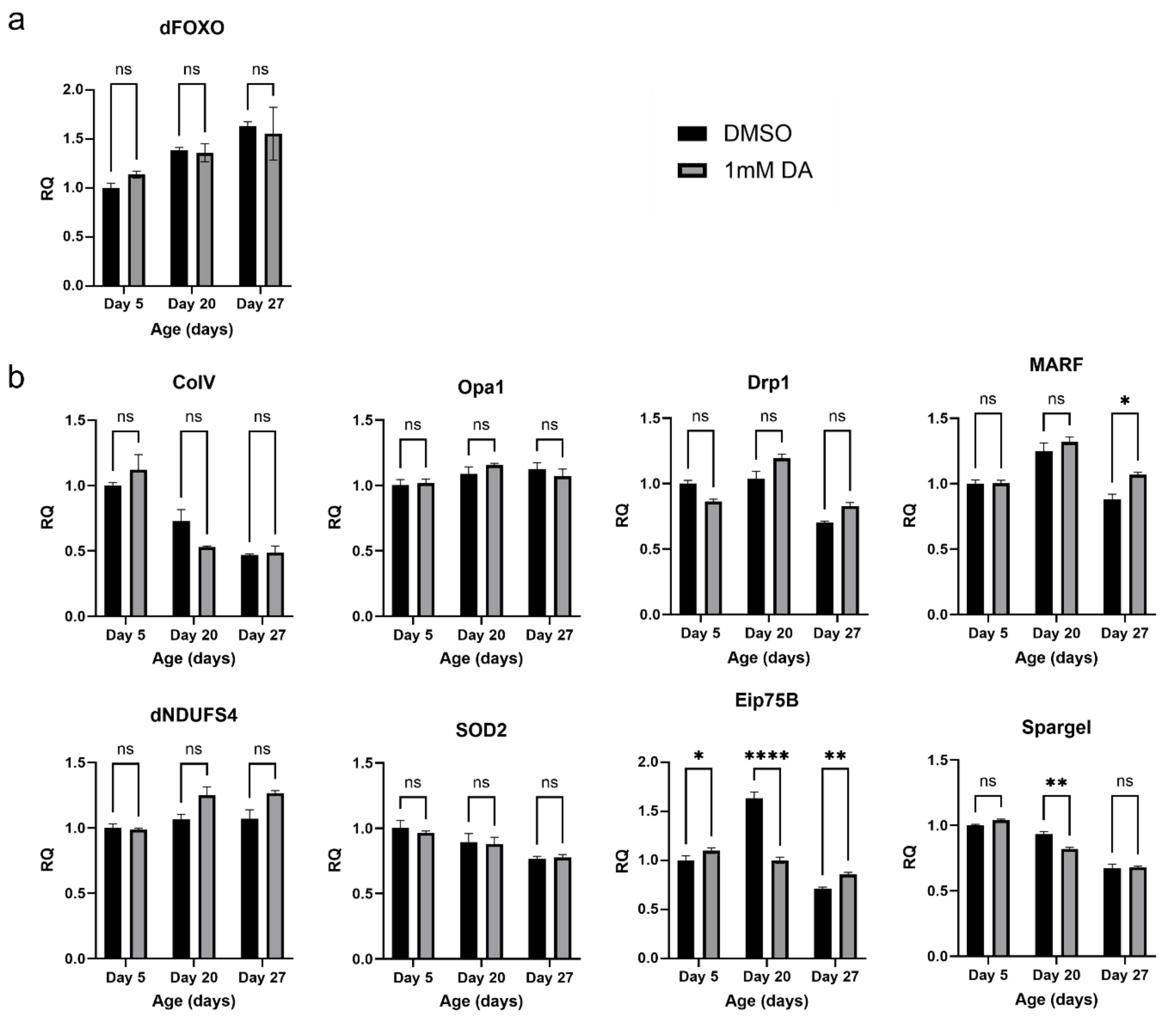

Fig. S4. DA has no significant effect on the expression of *dFOXO* and mitochondrial function-related genes, except for *Eip75B* in *iPLA-VIA*$^{\Delta 174}$. **a** Expression levels of *dFOXO* in *iPLA-VIA*$^{\Delta 174}$ flies at days 5, 20 and 27 post-eclosion fed with DMSO and 1 mM DA. DA does not affect *dFOXO* expression across all age groups and the treatment/age interaction is not significant ($p = 0.3158$). **b** The expression levels of mitochondrial function-related genes, including *CoIV*, *Opa1*, *MARF*, *dNDUFS4*, *SOD2*, and *Spargel*, were unaffected by DA treatment, with no significant interaction between treatment and age ($p = 0.0787$, $p = 0.3947$, $p = 0.0844$, $p = 0.052793$, $p = 0.8342$, respectively), except for *Eip75B* ($p = 0.0018$) and *Drp1* ($p = 0.000970$). Statistical analyses of all the RQ values were conducted using two-way ANOVA with Tukey's multiple comparisons tests in GraphPad Prism. Error bars represent SEM. * $p < 0.0332$, ** p 0.0021, **** $p < 0.0001$, n.s. = not significant (n = 3).

Disease Models & Mechanisms • Supplementary information

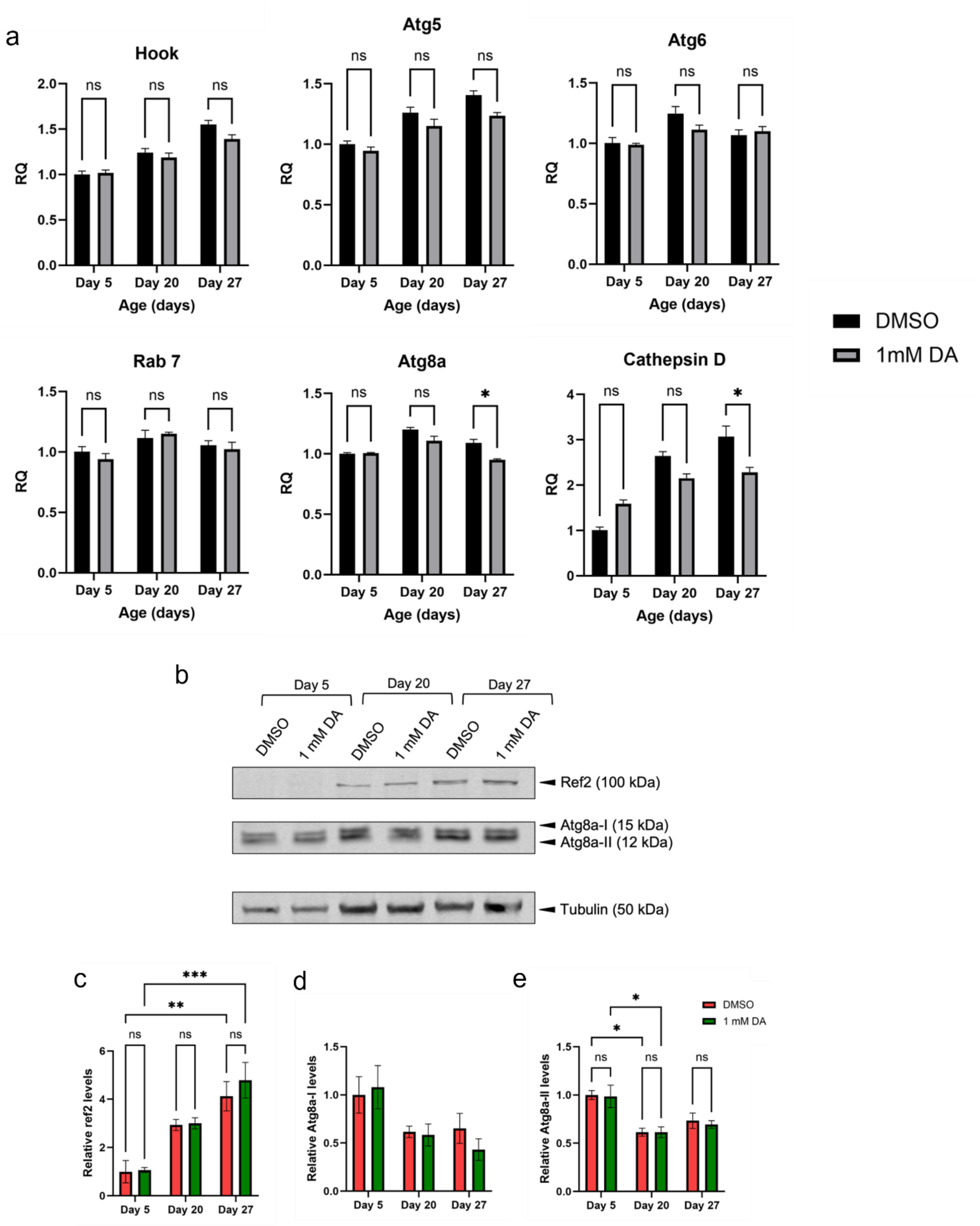

Fig. S5. DA treatment does not affect the expression of autophagy-related genes or the protein levels of Raf2 and Atg8a. **a** Expression level of *Hook, Atg5, Atg6, Rab7, Atg8a* and *Cathepsin D* showed no significant difference between DA-fed and solvent-treated flies. The interaction between treatment and age was not significant for most genes (*Hook*: p = 0.1807, *Atg5*: p = 0.3689, *Atg6*: p = 0.1708, *Rab7*: p = 0.5931), except for *Atg8a* (p = 0.0174) and *Cathepsin D* (p = 0.00396). Statistical analyses of all the RQ values were conducted using

Disease Models & Mechanisms • Supplementary information

two-way ANOVA with Tukey's multiple comparisons tests in GraphPad Prism. Error bars represent SEM., * $p < 0.0332$, n.s. = not significant (n = 3). **b** Western blot analyses of Ref2, Atg8a-I and Atg8a-II in whole fly extracts at days 5, 20 and 27 post-eclosion, treated with 1 mM decanoic acid (DA) or untreated (DMSO). Tubulin was used as a loading control, and relative expression levels were calculated based on tubulin expression. **c** Ref2 protein levels relative to tubulin increase with age, but treatment with 1 mM DA has no effect (n = 6). **d** Atg8a-I levels relative to tubulin remain unchanged with age or DA treatment (n = 6). **e** Atg8a-II levels relative to tubulin decrease with age, but DA treatment has no effect (n = 6). In all cases, $p < 0.05$ is considered statistically significant (* $p < 0.05$, ** $p < 0.01$, *** $p < 0.001$, n.s. = not significant). Comparisons not shown were not significant. In all cases, a two-way ANOVA revealed no statistically significant interactions between age and treatment.

Disease Models & Mechanisms • Supplementary information

Table S1. Sequences of the qPCR primers.

**Gene**	**Primer**
InR	Forward: AAGCGTGGGAAAATTAAGATGGA
	Reverse: GGCTGTCAACTGCTTCTACTG
d4E-BP	Forward: AAGATGTCCGCTTCACCCAC
	Reverse: TGGAGTAGAGGGTTCCGCC
dFOXO	Forward: CAGCAAACGGCGATCAACAA
	Reverse: CCTCGCCAGCCCAAAAGATA
dSir2	Forward: CATTATGCCGCATTTCGCCA
	Reverse: GAAGGTGTTCACTGAGGCCA
Hsp70	Forward: AATTGAGACCGCTGGAGGTG
	Reverse: GACAGATCGAAGGTGCCCAA
Eip75B	Forward: CATTACGGCGTGCATTCCTG
	Reverse: GTCCAAAACGCACAGCATCG
Spargel	Forward: GTTGCAATGCCGCAAATCT
	Reverse: GCCACCAGGGTGGAGTAG
Atg1	Forward: ACCAGAGGCAGAACGCATAC
	Reverse: GCAGCCAATTAGCGTAAAGC
Atg5	Forward: GACATCCAACCGCTCTGCGCA
	Reverse: CAGACGATGACTTCACGTACACC
Atg8a	Forward: CAATACAAGGAGCACGC
	Reverse: CGTGATGTTCCTGGTACAGGGA
Rab7	Forward: CAAACGCTTCTCCAACCAATAC
	Reverse: AGATCTGCATTGTGACCACTC
Atg6	Forward: GGAGTTATCTTTGCCCATC
	Reverse: TAGAGTCCGTAAGCCTGT
Hook	Forward: CGCATTCTTTCCCTGTCGCAT
	Reverse: GGAGTGTCTTTTGTTCATTTAGC
Cathepsin D	Forward: ATCATCGGTGGTCAGTATGT
	Reverse: AGAATATAGTCCTTGCCCTGC

Disease Models & Mechanisms • Supplementary information

CoIV	Forward: ATGGCCCTGCGACTACTCA
	Reverse: GTTGGTGGGCTCACGGAAG
MARF	Forward: GGCGAGGCGTATCTTATGAC
	Reverse: AGCTTCTCCTGGCACAA
Opa1	Forward: CTCTGAGCACCAAGCTAT
	Reverse: GGCGCAACTTGATGTCTA
Drp1	Forward: ATTGTTGTTCTAGGCAGTCAG
	Reverse: GAACTCTTGCCGGAGCT
dNDUFS4	Forward: AAGATCACCGTGCCGACTG
	Reverse: GACAATGGGTCGCCGCTG
SOD2	Forward: TGGCCACATCAACCACAC
	Reverse: TTCCACTGCGACTCGATG
iPLA-VIA	Forward: AAGCCGAGGAACGCTTCAAT
	Reverse: CCAGGAGGGATTATCAGCCAAG
